# Minigene-based characterization and classification of splice-associated variants in succinate dehydrogenase B

**DOI:** 10.1038/s41698-026-01685-7

**Published:** 2026-09-08

**Authors:** Anni Köhler, Alexandra A. Baumann, Natasha Lewis, Anja Richter, Susan Richter, Doreen William, Andrés Cruz-García, Hanno Glimm, Stefan Fröhling, Diana Le Duc, Evelin Schröck, Arne Jahn

**Affiliations:** 1 Institute for Clinical Genetics, University Hospital Carl Gustav Carus at TUD Dresden University of Technology and Faculty of Medicine of TUD Dresden University of Technology, Dresden, Germany; 2 National Center for Tumor Diseases (NCT), NCT/UCC Dresden, a partnership between German Cancer Research Center (DKFZ), Faculty of Medicine and University Hospital Carl Gustav Carus, TUD Dresden University of Technology, and Helmholtz-Zentrum Dresden-Rossendorf (HZDR), Dresden, Germany; 3 ERN-GENTURIS, Hereditary Cancer Syndrome Center, Dresden, Germany; 4 DKTK, Partner Site Dresden, Dresden, Germany; 5 Department of Systems Biology and Bioinformatics, University of Rostock, Rostock, Germany; 6 Institute for Medical Informatics and Biometry, Faculty of Medicine and University Hospital Carl Gustav Carus, TUD Dresden University of Technology, Dresden, Germany; 7 Auckland Cancer Society Research Centre, School of Medical Sciences, University of Auckland, Auckland, New Zealand; 8 Core Unit for Molecular Tumor Diagnostics (CMTD), NCT/UCC Dresden, a Partnership between German Cancer Research Center (DKFZ), Faculty of Medicine and University Hospital Carl Gustav Carus, TUD Dresden University of Technology and Helmholtz-Zentrum Dresden-Rossendorf (HZDR), Dresden, Germany; 9 Department for Translational Medical Oncology, National Center for Tumor Diseases (NCT/UCC) Dresden, a partnership between DKFZ, Faculty of Medicine and University Hospital Carl Gustav Carus, TUD Dresden University of Technology, and Helmholtz-Zentrum Dresden-Rossendorf (HZDR), Dresden, Germany; 10 Translational Medical Oncology, Faculty of Medicine and University Hospital Carl Gustav Carus, TUD Dresden University of Technology, Dresden, Germany; 11 Division of Translational Medical Oncology, German Cancer Research Center (DKFZ), Heidelberg, Germany; 12 National Center for Tumor Diseases (NCT), NCT Heidelberg, a partnership between DKFZ and Heidelberg University Hospital, Heidelberg, Germany; 13 German Cancer Consortium (DKTK), Core Center Heidelberg, Heidelberg, Germany; 14 Division of Translational Precision Medicine, Institute of Human Genetics, Heidelberg University, Heidelberg, Germany

**Keywords:** Cancer, Genetics, Oncology

## Abstract

Pathogenic germline variants in *SDHB* predispose to pheochromocytoma and paraganglioma, but limited functional evidence challenges clinical interpretation. To investigate splice-associated *SDHB* variants, we developed a minigene spanning exons 2-5 and assessed derived *SDHB* transcripts in HEK293T cells using targeted RNA sequencing. We evaluated 48 variants prioritized by SpliceAI (Δ≥0.42), two negative controls and endogenous *SDHB*, and compared findings with tumor data (*n* = 2). Nineteen variants (38%) showed ≥90% wildtype splicing, whereas 17 (34%) exhibited ≥90% aberrant splicing. Across all variants, 73 aberrant transcripts were observed (average of 2.3 per variant, 22 unique transcripts). Using a customized decision framework, RNA-based evidence strengths were assigned to 64 aberrant transcripts (88%). Among 26 classified variants, 10 received PVS1_Strong (RNA) (38%), including eight canonical splice-site variants, one missense variant and one stop-gain variant; two received PVS1_Moderate (RNA) and 14 received BP7_Strong (RNA). Integration of minigene RNA data changed ACMG scores by a mean of 2.7 points and led to reclassification of 13 variants (50%), including 12 downgrades from VUS to likely benign and one downgrade from likely pathogenic to VUS. These findings demonstrate that targeted sequencing of minigene-derived transcripts provides a scalable approach to evaluate splice-associated *SDHB* variants and improve variant classification.

## Introduction

Genetic testing is increasingly used in precision oncology to identify targetable vulnerabilities of tumors or individuals with hereditary cancer, but the functional classification of genetic variants remains challenging. Succinate dehydrogenase [ubiquinone] iron-sulfur subunit (*SDHB*) is a core component of the SDH complex or complex II, which uniquely links the tricarboxylic acid (TCA) cycle and the electron transport chain. Heterozygous pathogenic loss-of-function germline variants in *SDHB* and other tumor suppressor genes are observed in 24–44% of individuals with paraganglioma or pheochromocytoma (PPGL), rare tumors derived from neuroendocrine tissues. In addition to PPGLs, the risks for other cancers, such as gastrointestinal stromal tumors and renal cell carcinoma are increased in hereditary PPGL syndromes^1–3^. Moreover, *SDHB*-associated hereditary PPGL syndrome is frequently associated with aggressive disease behavior and poor clinical outcomes, underscoring the clinical importance of accurate and timely variant interpretation. As an allelic disorder, biallelic hypomorphic variants of *SDHB* are linked to mitochondrial complex II deficiency^4^.

Approximately 40% of *SDHB* variants are currently classified as variants of uncertain significance (VUS) in the ClinVar database^5^, highlighting a substantial gap for clinical decision making. To address this challenge, current efforts to improve classifications for endocrine tumor predisposition variants (ENDO-TPS VCEP), including *SDHB*, are ongoing and coordinated by the Clinical Genome Resource (ClinGen^6^). Nevertheless, reliable assessment of pathogenicity, especially for intronic and synonymous variants, is a non-trivial task and typically leads to a classification as VUS according to ACMG/AMP criteria^7^, as functional readouts and patient samples are rarely available. Although in silico tools, such as SpliceAI^8^ can help identify and prioritize potentially splice-disrupting variants, these predictions must be complemented by experimental assays. With the continuous expansion of genetic testing increasing the numbers of variants, there is a growing need for scalable approaches capable of classifying large numbers of variants.

One well-established and streamlined assay to interrogate splice-associated variants is the minigene assay, which investigates the impact of genetic variants on pre-mRNA splicing. By cloning genomic fragments covering the exons of interest and their flanking intronic sequences into a plasmid, variant-induced splicing changes can be directly assessed in a controlled cellular context. Minigene assays have been successfully applied to tumor suppressor genes such as *CHEK2, RAD51C, BRCA1, BRCA2, TP53, MLH1, MSH2, MSH6*, and *PMS2*^9–14^, thereby improving variant classification and clinical translation.

In this study, we established and validated an *SDHB* minigene to systematically assess the impact of 48 variants, located in exon 3, 4, and adjacent intronic regions, on splicing. Variants were prioritized with SpliceAI^8^ and a targeted NGS-based workflow was implemented to enable precise identification, characterization, and quantification of transcripts. Additionally, we integrated RNA-based insights into an adapted ACMG/AMP framework to support *SDHB* variant reclassification. Together, this approach provides a quantitative and scalable strategy to improve classification of splice-associated variants in *SDHB*.

## Results

### Minigene construction and variant selection

**Fig. 1:**
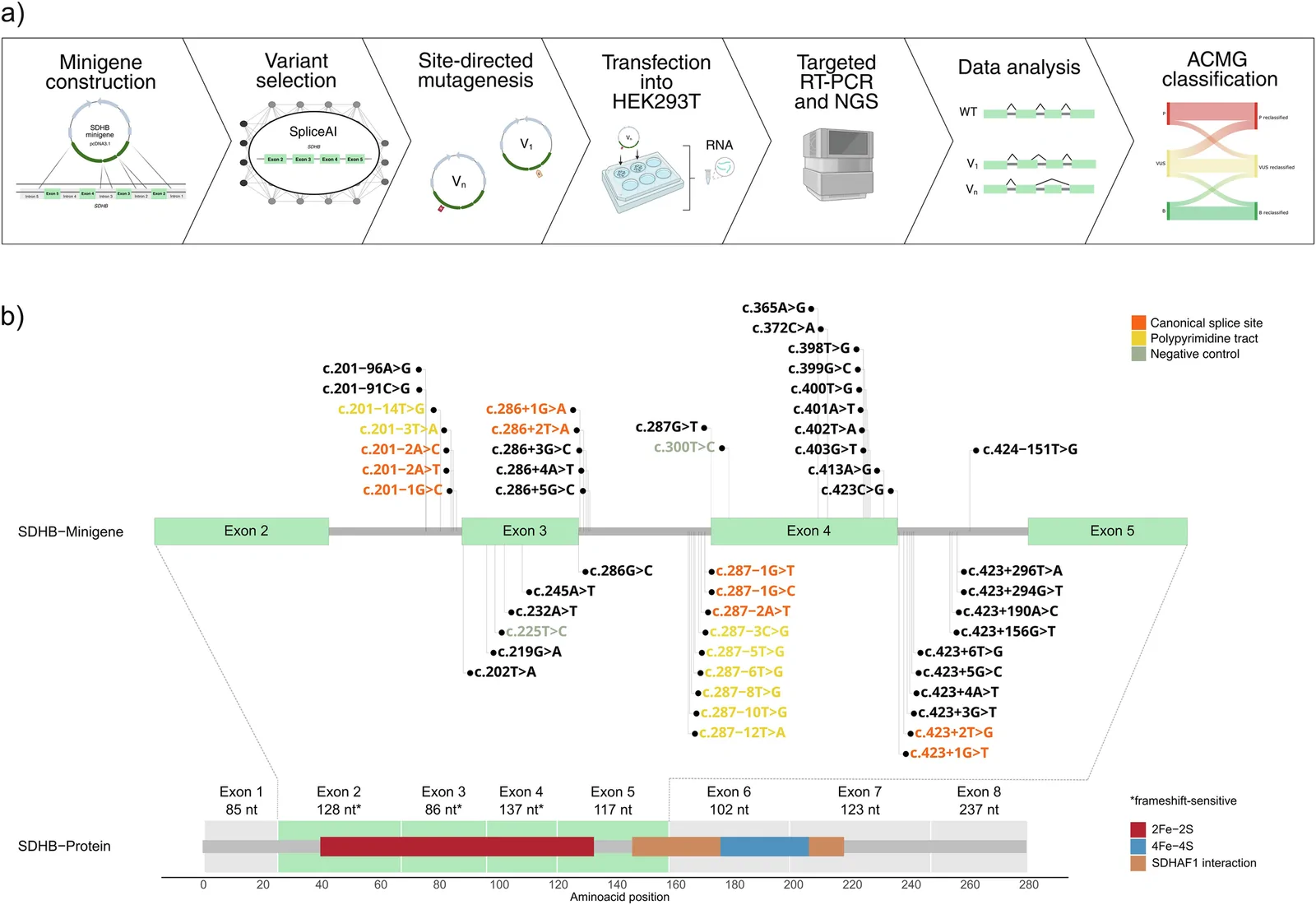
Workflow and variant selection to identify aberrant splicing of in silico prioritized variants with an *SDHB* minigene of exons 2-5. **a** Workflow representing the *SDHB* minigene construction, variant prioritization, introduction of variants into the minigene, assessment of splicing with next-generation sequencing (NGS) and variant reclassification. RT-PCR, Reverse transcription polymerase chain reaction. (Created in BioRender. Köhler, A. (2026)). **b** Lollipop plot showing the distribution of 48 *SDHB* single nucleotide variants prioritized with SpliceAI and analyzed with the minigene comprising exons 2–5 and neighboring intronic sequences. Annotated splicing elements are indicated, including canonical splice sites (orange) and polypyrimidine tracts (yellow). Negative control variants are highlighted in green (Supplementary Data 1). The sequence is mapped to the *SDHB* transcript NM_003000.3 and annotated with exon numbers, amino acid positions and functional protein domains from UniProt P21912 reference. 2Fe-2S and 4Fe-4S refer to iron sulfur clusters. (Created with R).

Based on the functionally relevant iron sulfur cluster domains, the exonic reading frame and limitations of plasmid size, we selected exons 2–5 with adjacent intronic sequences as region of interest for a partial *SDHB* minigene. This region was cloned into the mammalian expression vector pcDNA3.1 (Fig. 1a, and Supplementary Fig. 1).

**Fig. 2:**
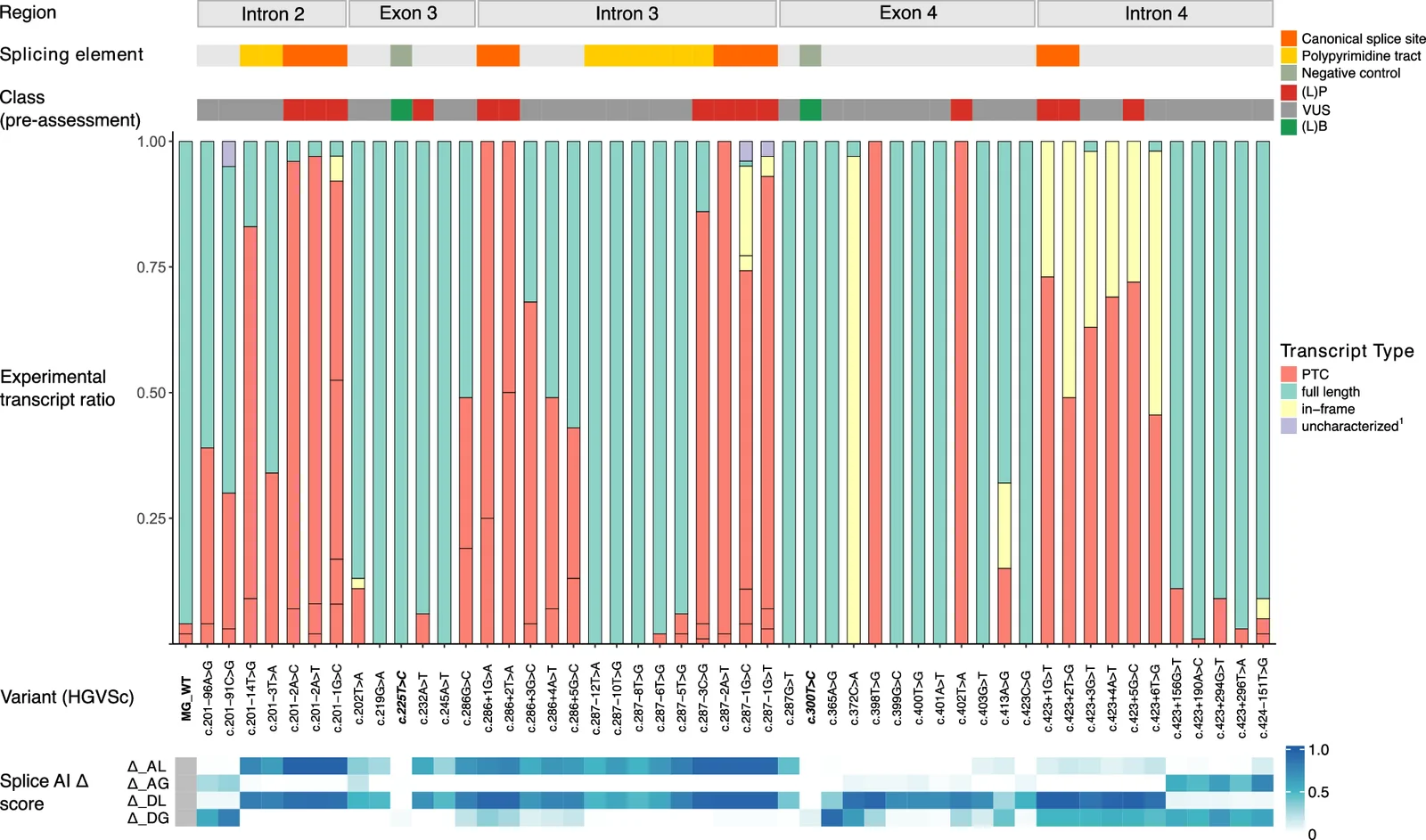
Experimental transcript outcomes and SpliceAI predictions for 50 *SDHB* variants. Overview of the analyzed genomic regions (intron 2 to intron 4) with annotated splicing elements, including canonical splice sites (orange), polypyrimidine tracts (yellow) and negative controls (green, HGVSc highlighted in bold italics). The pre-assessment variant classification prior to integration of RNA evidence was indicated ((L)P, likely pathogenic/pathogenic; VUS, variant of uncertain significance; (L)B, likely benign/benign). Stacked bar plots show the distribution of transcripts assessed by minigene assay for each variant, ratios normalized to 1.0, classified as premature termination codon (PTC, red), full-length (teal), in-frame (yellow), or uncharacterized transcripts (purple). Variants were labeled using HGVS cDNA nomenclature and ordered by genomic position. The lower heatmap displays unmasked SpliceAI delta scores (AL, acceptor loss; AG, acceptor gain; DL, donor loss; DG, donor gain), with color intensity indicating predicted splicing impact. Footnotes: ^1^Low-abundance aberrant transcripts (<5%) indicating intron retention events that could not be reliably interpreted because of the shortened flanking intronic sequence in the minigene. (Created with R).

We prioritized putative splice-associated variants in this region with the in silico prediction tool SpliceAI^8^, which demonstrated high predictive accuracy and sensitivity^15,16^. From 481 single nucleotide variants (SNVs) with at least one SpliceAI ∆-score above 0.2 (Supplementary Data 1), we prioritized 48 variants with a maximum ∆-score above 0.42 (range: 0.42–0.97, mean 0.76) (Fig. 1b). SpliceAI predicted acceptor gain for five variants, donor gain for 15 variants, donor-loss for 17 variants, acceptor-loss for one variant and both acceptor and donor loss for 21 variants (all Δ ≥0.42) (Fig. 2). Ten variants were located at canonical splice sites (donor or acceptor of exons 3 or 4), 16 were exonic and 22 were intronic, including eight variants assigned to the polypyrimidine tract. Two additional variants (c.225T>C and c.300T>C) were included as negative controls based on ClinVar and population frequencies (gnomAD^17^), yielding a total of 50 variants. Variant assessment prior to minigene testing yielded two variants as (likely) benign, 14 variants as (likely) pathogenic and 34 variants as variants of uncertain significance. Prioritized variants were introduced in the vector construct to create 50 minigenes with one SNV each. Wildtype and mutant minigenes were transfected into HEK293T cells and analyzed with a targeted NGS workflow quantifying splice junctions (relative abundance ≥1%) (Fig. 1a).

### Transcript analysis of endogenous and minigene derived *SDHB*

To assess *SDHB* splicing in HEK293T, we performed transcript analysis of endogenous *SDHB*, which revealed a single isoform corresponding to the MANE transcript NM_003000.3 (Supplementary Fig. 3, and Supplementary Data 2). Introduction of the wild-type minigene resulted in mRNA corresponding almost completely to the MANE transcript (96%, NM_003000.3). Only very low fractions of exon 3 skipping (out of frame, 2%) and partial exon 4 skipping (out of frame, 2%) were observed (Fig. 2, and Supplementary Data 3). In addition, low levels (<1%) of a 54-nucleotide in-frame partial exon 4 skipping were detected, corresponding to the *SDHB* isoform ENST00000485515.6. This isoform is ubiquitously expressed at very low levels across tissues (typically <1 TPM) according to GTEx data^18^. Based on these findings, endogenous *SDHB* in HEK293T and *SDHB* minigene-derived transcript profile closely resembled splicing of biologically relevant *SDHB* transcripts, supporting its suitability for investigating the transcriptional impact of in silico predicted splice-associated variants.

While 14 out of 50 variants (28.0%), including two negative controls, exclusively showed wildtype transcript, at least one aberrant transcript was detected for 36 variants (72.0%) (Fig. 2). The average number of observed transcripts per variant was 2.3 (median two, range one to six). Across all variants, we observed 113 transcripts in total, including full-length wildtype-transcripts for 40 variants and 73 aberrant transcripts for 36 variants. These transcripts corresponded to 22 unique aberrant transcripts (Supplementary Data 3). Classification of the 22 unique aberrant transcripts by splice effect revealed partial exon skipping (40.9%, *n* = 9 unique transcripts across 18 variants), pseudo-exon inclusion (27.3%, 6 unique transcripts across 6 variants), intron retention (18.2%, 4 unique transcripts across 10 variants), single exon skipping (9.1%, *n* = 2 unique transcripts across 19 variants) and multi-exon skipping (4.5%, 1 unique transcript across 5 variants) (Supplementary Figs. 4 and 5). While 19.2% of all aberrant transcripts (*n* = 14 of 73 across 13 variants) preserved the reading frame, 80.8% of aberrant transcripts (*n* = 59 of 73 across 35 variants) led to an expected premature termination codon (PTC) (Fig. 2, and Supplementary Data 3). Among the 34 variants leading to ≥2 aberrant transcripts, 26 were found to express both aberrantly spliced transcript(s) and wildtype-transcript. Three additional aberrant transcripts could not be fully characterized, as they represented whole intron retention events within the minigene at low relative abundance (<5%). As the minigene construct contains only limited flanking intronic sequence, these events could not be reliably evaluated. Among 48 variants prioritized by SpliceAI (Δ ≥0.42), 17 (35%) exhibited ≥90% aberrant splicing, confirming splice disruption. In contrast, 19 variants (40%) with an unmasked SpliceAI Δ score ≥0.42 showed ≥90% wild-type expression, indicating preserved splicing despite high in silico splice disruption scores (Fig. 2). For five of these variants (one synonymous and four missense variants) the masked SpliceAI score was below 0.2 (Table 1, and Supplementary Data 1). An additional five variants located near the minigene boundaries were tested, but splicing outcomes could not be reliably assessed due to the absence of adjacent splice sites within the construct (Supplementary Data 3, “no functional assessment”).

**Table 1:** Integration of variant-specific splicing outcomes into ACMG/AMP classification for *SDHB* variants

Variant selection					Transcript analysis		ACMG classification with RNA data			
Variant (HGVSc)	HGVSp*	Class*	Δscore max (unmasked)	Δscore max (masked)	PVS1_Strength (RNA)	Combined strength	Overall RNA strength	ACMG codes with RNA strength	ACMG points with RNA strength	ACMG class with RNA strength
*c.225T*>*C*	*p.Ala75*=	*LB*	*0.03*	*0.03*	*Negative control*	*BP7_S (1.00)*	*BP7_S (RNA)*	*PM2 Supporting* *BS2 Supporting* *BP7 Strong (RNA)*	*-4*	*LB*
*c.300T*>*C*	*p.Ser100*=	*B*	*0.09*	*0.09*	*Negative control*	*BP7_S (1.00)*	*BP7_S (RNA)*	*BS1 Strong* *BS2 Strong* *BP7 Strong (RNA)*	*-12*	*B*
c.201-96A>G	p.?	VUS	0.57	0.57	PVS1: 0.35 (r.200_201ins[201-211_201-97], p.(Cys68AsnTer6)); 0.04 (r.200_201ins[201-226_201-97], p.(Lys67AsnTer12))	PVS1 (0.39) + BP7_S (0.61)	PVS1_N/A (RNA)^d^	not applicable^d^	N/A	N/A
c.201-91C>G	p.?	VUS	0.81	0.81	PVS1: 0.27 (r.200_201ins[201-211_201-92], p.(Cys68AsnTer6)); 0.03 (r.200_201ins[201-226_201-92], p.(Lys67AsnTer12))	PVS1 (0.30) + BP7_S (0.65) + N/A (0.05)^b^	PVS1_N/A (RNA)^d^	not applicable^d^	N/A	N/A
c.201-14T>G	p.?	VUS	0.82	0.76	PVS1: 0.74 (r.201_286del, p.(Cys68HisfsTer21)); 0.09 (r.200_201ins[201-13_201-1], p.(Lys67AsnfsTer4))	PVS1 (0.83) + BP7_S (0.17)	PVS1_Moderate (RNA)	PM2 Supporting PVS1 Moderate (RNA)	3	VUS
c.201-3T>A	p.?	VUS	0.8	0.64	PVS1: 0.34 (r.201_286del, p.(Cys68HisfsTer21))	PVS1 (0.34) + BP7_S (0.66)	PVS1_N/A (RNA)^d^	not applicable^d^	N/A	N/A
c.201-2A>C	p.?	P	0.97	0.97	PVS1: 0.89 (r.201_286del, p.(Cys68HisfsTer21)); 0.06 (r.201_234del, p.(Cys68LeufsTer8)); 0.02 (r.200_201ins[201-4_201-1], p.(Lys67AsnfsTer14))	PVS1 (0.97) + BP7_S (0.03)	PVS1_Strong (RNA)	PVS1 Strong (RNA) PS1 Strong PM2 Supporting PS4 Moderate	11	P
c.201-2A>T	p.?	P	0.97	0.97	PVS1: 0.89 (r.201_286del, p.(Cys68HisfsTer21)); 0.07 (r.201_234del, p.(Cys68LeufsTer8))	PVS1 (0.96) + BP7_S (0.04)	PVS1_Strong (RNA)	PVS1 Strong (RNA) PS1 Strong PM2 Supporting	9	LP
c.201-1G>C	p.?	P	0.97	0.97	PVS1: 0.40 (r.201_202del, p.(Cys68TrpfsTer11)); 0.36 (r.201_234del, p.(Cys68LeufsTer8)); 0.09 (r.200_201ins[201-4_201-1], p.(Lys67AsnfsTer14)); 0.08 (r.201_286del, p.(Cys68HisfsTer21)) PVS1_M: 0.05 (r.201_206del, p.(Lys67_Gly69del))	PVS1 (0.93) + PVS1_M (0.05) + BP7_S (0.03)	PVS1_Strong (RNA)	PVS1 Strong (RNA) PS1 Strong PM2 Supporting	9	LP
c.202T>A	p.Cys68Ser	VUS	0.5	0.34	PVS1: 0.11 (r.201_286del, p.(Cys68HisfsTer21)) PVS1_M: 0.02 (r.201_203del, p.(Lys67_Cys68del))	PVS1 (0.11) + PVS1_M (0.02) + BP7_S (0.87)	PVS1_N/A (RNA)^d^	not applicable^d^	N/A	N/A
c.219G>A	p.Leu73=	VUS	0.48	0.27	–	BP7_S (1.00)	BP7_S (RNA)	PM2 Supporting PP3 Supporting BP7 Strong (RNA)	-2	LB
c.232A>T	p.Lys78Ter	LP	0.71	0.57	PVS1: 0.06 (r.201_286del, p.(Cys68HisfsTer21))	PVS1 (0.06) + BP7_S (0.94)	BP7_S (RNA) not applicable^e^	not applicable^e^	N/A	N/A
c.245A>T	p.Glu82Val	VUS	0.47	0.47	-	BP7_S (1.00)	BP7_S (RNA) not applicable^e^	not applicable^e^	N/A	N/A
c.286G>C	p.Gly96Arg	VUS	0.8	0.8	PVS1: 0.30 (r.201_286del, p.(Cys68HisfsTer21)); 0.19 (r.286_287ins[286 + 1_286 + 144], p.(Gly96ArgfsTer37))	PVS1 (0.49) + BP7_S (0.51)	PVS1_N/A (RNA)^d^	not applicable^d^	N/A	N/A
c.286+1G>A	p.?	P	0.94	0.94	PVS1: 0.75 (r.286_287ins[286 + 1_286 + 144], p.(Gly96AspfsTer37)); 0.25 (r.201_286del, p.(Cys68HisfsTer21))	PVS1 (1.00)	PVS1_Strong (RNA)	PVS1 Strong (RNA) PS1 Strong PM2 Supporting PS4 Moderate	11	P
c.286+2T>A	p.?	P	0.94	0.94	PVS1: 0.50 (r.201_286del, p.(Cys68HisfsTer21)); 0.50 (r.286_287ins[286 + 1_286 + 144], p.(Ile97GlufsTer36))	PVS1 (1.00)	PVS1_Strong (RNA)	PVS1 Strong (RNA) PS1 Strong PM2 Supporting PS4 Moderate	11	P
c.286+3G>C	p.?	VUS	0.67	0.67	PVS1: 0.64 (r.201_286del, p.(Cys68HisfsTer21)); 0.04 (r.286_287ins[286 + 1_286 + 144], p.(Ile97GlnfsTer36))	PVS1 (0.68) + BP7_S (0.32)	PVS1_N/A (RNA)^d^	not applicable^d^	N/A	N/A
c.286+4A>T	p.?	VUS	0.78	0.78	PVS1: 0.42 (r.201_286del, p.(Cys68HisfsTer21)); 0.07 (r.286_287ins[286 + 1_286 + 144], p.(Ile97ValfsTer36))	PVS1 (0.49) + BP7_S (0.51)	PVS1_N/A (RNA)^d^	not applicable^d^	N/A	N/A
c.286+5G>C	p.?	VUS	0.78	0.78	PVS1: 0.30 (r.201_286del, p.(Cys68HisfsTer21)); 0.13 (r.286_287ins[286 + 1_286 + 144], p.(Ile97AspfsTer36))	PVS1 (0.43) + BP7_S (0.57)	PVS1_N/A (RNA)^d^	not applicable^d^	N/A	N/A
c.287-12T>A	p.?	VUS	0.51	0.51	–	BP7_S (1.00)	BP7_S (RNA)	PM2 Supporting PP3 Supporting BP7 Strong (RNA)	-2	LB
c.287-10T>G	p.?	VUS	0.65	0.65	–	BP7_S (1.00)	BP7_S (RNA)	PM2 Supporting PP3 Supporting BP7 Strong (RNA)	-2	LB
c.287-8T>G	p.?	VUS	0.51	0.51	–	BP7_S (1.00)	BP7_S (RNA)	PM2 Supporting PP3 Supporting BP7 Strong (RNA)	-2	LB
c.287-6T>G	p.?	VUS	0.67	0.67	PVS1: 0.02 (r.287_423del, p.(Ile97PhefsTer11))	PVS1 (0.02) + BP7_S (0.98)	BP7_S (RNA)	PM2 Supporting PP3 Supporting BP7 Strong (RNA)	-2	LB
c.287-5T>G	p.?	VUS	0.81	0.81	PVS1: 0.04 (r.287_423del, p.(Ile97PhefsTer11)); 0.02 (r.201_423del, p.(Cys68IleTer2))	PVS1 (0.06) + BP7_S (0.94)	BP7_S (RNA)	PM2 Supporting PP3 Supporting BP7 Strong (RNA)	-2	LB
c.287-3C>G	p.?	LP	0.94	0.94	PVS1: 0.82 (r.287_423del, p.(Ile97PhefsTer11)); 0.03 (r.201_423del, p.(Cys68IleTer2)); 0.01 (r.287_411del, p.(Ile97SerfsTer15))	PVS1 (0.86) + BP7_S (0.14)	PVS1_Moderate (RNA)	PM2 Supporting PS4 Moderate PVS1 Moderate (RNA) PS1 Moderate	7	LP
c.287-2A>T	p.?	P	0.95	0.94	PVS1: 0.98 (r.287_423del, p.(Ile97PhefsTer11)); 0.02 (r.201_423del, p.(Cys68IleTer2))	PVS1 (1.00)	PVS1_Strong (RNA)	PVS1 Strong (RNA) PS1 Strong PM2 Supporting	9	LP
c.287-1G>C	p.?	P	0.95	0.94	PVS1: 0.86 (r.287_423del, p.(Ile97PhefsTer11)); 0.04 (r.201_423del, p.(Cys68IleTer2)); 0.03 (r.287_411del, p.(Ile97SerfsTer15)) PVS1_S: 0.04 (r.287_322del, p.(Ile97_Gly108del))	PVS1 (0.93) + PVS1_S (0.04) + N/A (0.03)^c^	PVS1_Strong (RNA)	PVS1 Strong (RNA) PS1 Strong PM2 Supporting PS4 Moderate	11	P
c.287-1G>T	p.?	P	0.95	0.94	PVS1: 0.64 (r.287_423del, p.(Ile97PhefsTer11)); 0.07 (r.287_411del, p.(Ile97SerfsTer15)); 0.04 (r.201_423del, p.(Cys68IleTer2)) PVS1_S: 0.18 (r.287_322del, p.(Ile97_Gly108del)); 0.03 (r.287_334del, p.(Gly96_Leu111del))	PVS1 (0.75) + PVS1_S (0.21) + BP7_S (0.01) + N/A (0.04)^c^	PVS1_Strong (RNA)^f^	PVS1 Strong (RNA) PS1 Strong PM2 Supporting	9	LP
c.287G>T	p.Gly96Val	VUS	0.44	0.44	–	BP7_S (1.00)	BP7_S (RNA) not applicable^e^	not applicable^e^	N/A	N/A
c.365A>G	p.Asn122Ser	VUS	0.92	0.92	–	BP7_S (1.00)	BP7_S (RNA) not applicable^e^	not applicable^e^	N/A	N/A
c.372C>A	p.Val124=	VUS	0.88	0.88	PVS1_N/A: 0.97 (r.370_423del, p.(Val124_Pro141del))^a^	BP7_S (0.03) + PVS1_N/A (0.97)^a^	PVS1_N/A^a^	not applicable^a^	N/A	N/A
c.398T>G	p.Met133Arg	VUS	0.94	0.94	PVS1: 1.00 (r.399_423del, p.(Met133ArgfsTer2))	PVS1 (1.00)	PVS1_Strong (RNA)	PM2 Supporting PVS1 Strong (RNA)	5	VUS
c.399G>C	p.Met133Ile	VUS	0.73	0.05^¥^	–	BP7_S (1.00)	BP7_S (RNA) not applicable^e^	not applicable^e^	N/A	N/A
c.400T>G	p.Tyr134Asp	VUS	0.73	0.07^¥^	–	BP7_S (1.00)	BP7_S (RNA) not applicable^e^	not applicable^e^	N/A	N/A
c.401A>T	p.Tyr134Phe	VUS	0.72	0.02^¥^	–	BP7_S (1.00)	BP7_S (RNA) not applicable^e^	not applicable^e^	N/A	N/A
c.402T>A	p.Tyr134Ter	LP	0.81	0.81	PVS1: 1.00 (r.399_423del, p.(Tyr134IlefsTer1))	PVS1 (1.00)	PVS1_Strong (RNA)	PVS1 Strong (RNA) PM2 Supporting	5	VUS
c.403G>T	p.Val135Leu	VUS	0.72	0.13^¥^	–	BP7_S (1.00)	BP7_S (RNA) not applicable^e^	not applicable^e^	N/A	N/A
c.413A>G	p.Asp138Gly	VUS	0.42	0.42	PVS1: 0.15 (r.399_423del, p.(Tyr134IlefsTer1)) PVS1_N/A: 0.17 (r.370_423del, p.(Val124_Pro141del))^a^	PVS1 (0.15) + BP7_S (0.68) + PVS1_N/A (0.17)^a^	PVS1_N/A^a^	not applicable^a^	N/A	N/A
c.423C>G	p.Pro141=	VUS	0.52	0.06^¥^	–	BP7_S (1.00)	BP7_S (RNA)	PM2 Supporting PP3 Supporting BP7 Strong (RNA)	-2	LB
c.423+1G>T	p.?	P	0.97	0.97	PVS1: 0.73 (r.399_423del, p.(Tyr134IlefsTer1)) PVS1_N/A: 0.27 (r.370_423del, p.(Val124_Pro141del))^a^	PVS1 (0.73) + PVS1_N/A (0.27)^a^	PVS1_N/A^a^	not applicable^a^	N/A	N/A
c.423+2T>G	p.?	P	0.97	0.97	PVS1: 0.49 (r.399_423del, p.(Tyr134IlefsTer1)) PVS1_N/A: 0.51 (r.370_423del, p.(Val124_Pro141del))^a^	PVS1 (0.49) + PVS1_N/A (0.51)^a^	PVS1_N/A^a^	not applicable^a^	N/A	N/A
c.423+3G>T	p.?	VUS	0.87	0.87	PVS1: 0.63 (r.399_423del, p.(Tyr134IlefsTer1)) PVS1_N/A: 0.35 (r.370_423del, p.(Val124_Pro141del))^a^	PVS1 (0.63) + BP7_S (0.02) + PVS1_N/A (0.35)^a^	PVS1_N/A^a^	not applicable^a^	N/A	N/A
c.423+4A>T	p.?	VUS	0.96	0.96	PVS1: 0.69 (r.399_423del, p.(Tyr134IlefsTer1)) PVS1_N/A: 0.31 (r.370_423del, p.(Val124_Pro141del))^a^	PVS1 (0.69) + PVS1_N/A (0.31)^a^	PVS1_N/A^a^	not applicable^a^	N/A	N/A
c.423+5G>C	p.?	LP	0.97	0.97	PVS1: 0.72 (r.399_423del, p.(Tyr134IlefsTer1)) PVS1_N/A: 0.28 (r.370_423del, p.(Val124_Pro141del))^a^	PVS1 (0.72) + PVS1_N/A (0.28)^a^	PVS1_N/A^a^	not applicable^a^	N/A	N/A
c.423+6T>G	p.?	VUS	0.89	0.89	PVS1: 0.46 (r.399_423del, p.(Tyr134IlefsTer1)) PVS1_N/A: 0.53 (r.370_423del, p.(Val124_Pro141del))^a^	PVS1 (0.46) + BP7_S (0.02) + PVS1_N/A (0.53)^a^	PVS1_N/A^a^	not applicable^a^	N/A	N/A
c.423+156G>T	p.?	VUS	0.56	0.56	PVS1: 0.11 (r.423_424ins[423 + 161_423 + 288], p.(Asp142GluTer35))	PVS1 (0.11) + BP7_S (0.89)	BP7_S (RNA)^g^	PM2 Supporting PP3 Supporting BP7 Strong (RNA)	-2	LB
c.423+190A>C	p.?	VUS	0.51	0.51	PVS1: 0.01 (r.423_424ins[423 + 161_423 + 288], p.(Asp142GluTer35))	PVS1 (0.01) + BP7_S (0.99)	BP7_S (RNA)	PM2 Supporting PP3 Supporting BP7 Strong (RNA)	-2	LB
c.423+294G>T	p.?	VUS	0.67	0.67	PVS1: 0.09 (r.423_424ins[423 + 161_423 + 288], p.(Asp142GluTer35))	PVS1 (0.09) + BP7_S (0.91)	BP7_S (RNA)	PM2 Supporting PP3 Supporting BP7 Strong (RNA)	-2	LB
c.423+296T>A	p.?	VUS	0.52	0.52	PVS1: 0.03 (r.423_424ins[423 + 161_423 + 293], p.(Asp142GluTer35))	PVS1 (0.03) + BP7_S (0.97)	BP7_S (RNA)	PM2 Supporting PP3 Supporting BP7 Strong (RNA)	-2	LB
c.424-151T>G	p.?	VUS	0.77	0.77	PVS1: 0.03 (r.399_423del, p.(Tyr134IlefsTer1)); 0.02 (r.423_424ins[424-150_424-1], p.(Asp142IlefsTer18)) PVS1_N/A: 0.04 (r.370_423del, p.(Val124_Pro141del))^a^	PVS1 (0.05) + BP7_S (0.91) + PVS1_N/A (0.04)^a^	BP7_S (RNA)	PM2 Supporting PP3 Supporting BP7 Strong (RNA)	-2	LB

Overview of all tested *SDHB* variants (*n* = 50) with identified transcripts and fractions, HGVS nomenclature, maximum SpliceAI Δ-scores and ACMG classifications for variants with conclusive transcript data with and without minigene derived RNA code. Asterisks in the column headers indicate that HGVSp and classification are based on the pre-assessment performed before integration of minigene-derived RNA evidence. The two negative control variants are listed at the top of the table; all remaining variants are sorted in ascending order according to their coding DNA position. Variants with a masked SpliceAI Δ-score below 0.2 are labeled with ^¥^. Extended SpliceAI Δ-scores are provided in Supplementary Data 1. An extended version of this table, including ACMG evidence codes and point assignments prior to RNA evidence integration, is provided in Supplementary Data 4.

^a^Transcript corresponds to the alternative *SDHB* isoform ENST00000485515.6; overall RNA strength was not assigned if abundance was ≥10%.

^b^Total intron 2 retention (r.200_201ins[200+1_201-1], p.(Cys68Ter)).

^c^Total intron 3 retention (r.286_287ins[286+1_287-1], p.(Ile97GlufsTer36)).

^d^Overall RNA strength was not assigned because transcript(s) did not meet predefined interpretation thresholds.

^e^No aberrant splicing was observed in the splicing assay; however, BP7 was not applied due to a potential missense effect. FL_WT, full-length wildtype transcript; PVS1_S, strong PVS1 strength; PVS1_M, moderate PVS1 strength; PVS1_N/A, PVS1 not assigned; BP7_S, strong BP7 strength; (L)P, (likely) pathogenic; VUS, variant of uncertain significance; (L)B, (likely) benign.

^f^PVS1_Strong (RNA) overall strength assigned due to overall high percentage of splice disruption.

^g^BP7_S ratio rounded up.

The comparison of targeted NGS-based transcript analysis with conventional gel electrophoresis followed by Sanger sequencing of visible and excised bands revealed a clear advantage of the NGS-approach. Only 47% of transcripts (53 of 113) identified by NGS could be detected by gel electrophoresis and Sanger sequencing, particularly failing to capture small splicing alterations and unexpected effects (Supplementary Fig. 6). In addition, this alternative approach did not allow reliable transcript identification and quantification (Supplementary Figs. 6–8). Taken together, the *SDHB* minigene assay proved suitable for splicing analysis and enabled experimental confirmation of aberrant splicing for nearly three quarters of in silico prioritized variants.

### Integration of RNA analysis into ACMG/AMP variant classification

To integrate transcriptional insights into a weighted PVS1/BP7 (RNA) ACMG code for variant classification according to Tayoun and Walker^19,20^, we adapted the PVS1 decision tree to the gene and assay (Supplementary Fig. 2a). All aberrant transcripts leading to a premature termination codon (PTC) and thereby predicted to result in loss of function (LoF) received a PVS1_Strong code. In-frame transcripts predicted to disrupt functionally critical regions were likewise assigned a PVS1_Strong code (Supplementary Fig. 2a, 2b). Accordingly, a PVS1_strength code was assigned to 64 aberrant transcripts (Table 1, and Supplementary Data 3) and yielded 59 times PVS1 (92%), three times PVS1_Strong (5%) and twice PVS1_Moderate (3%). BP7_Strong was assigned to full-length wild-type transcripts in 40 variants. PVS1_Strong was given to three in-frame transcripts leading to partial deletions within the functionally critical 2Fe-2S ferredoxin-type domain (Pfam Fer2_3). PVS1_Moderate was assigned to two variants that led to small in-frame deletions within the Fer2_3 domain (Supplementary Fig. 2b). Nine transcripts could not be assigned a strength code (PVS1_N/A), all corresponding to an in-frame 54-nt partial exon 4 skipping (r.370_423del) matching the minor transcript isoform ENST00000485515.6 detected across multiple normal tissues^18^ (Supplementary Fig. 2b).

In addition, we developed a complementary decision tree to combine PVS1 and BP7 evidence, allowing assignment of an overall weighted PVS1 and BP7 (RNA) strength based on the observed transcriptional impact and relative transcript fraction (Supplementary Fig. 2c). An overall PVS1_strength (RNA) or BP7_Strong (RNA) was assigned per variant, with minigene-derived PVS1_strength code proportionally downweighted to a conservative maximum of strong (Supplementary Fig. 2d). For variants with multiple transcripts, individual transcript-level codes were combined using conservative thresholds (≥80% loss-of-function transcripts for PVS1_strength (RNA) (BRCA1/2 VCEP guidelines^21^), ≥90% wildtype transcript for BP7_Strong (RNA)) to derive an overall RNA strength (Table 1, and Supplementary Fig. 2c). This approach yielded a total of PVS1_Strong (RNA) codes for 10 variants (including eight canonical splice-site variants), PVS1_Moderate (RNA) codes for two variants, and BP7_Strong (RNA) codes for 14 intronic or synonymous variants (Table 1). For the eight missense variants with a SpliceAI Δ ≥0.42 but ≥90% wild-type transcript, we did not apply BP7_Strong (RNA), as a deleterious coding impact of the missense variant could not be ruled out. In addition, eight variants with complex splicing effects did not reach the predefined thresholds for overall RNA strength. Another eight variants (including two canonical splice-site variants) yielding ≥10% of the alternative splice product r.370_423del (ENST00000485515.6) were not assigned an overall RNA strength as a conservative measure given the uncertain protein impact of this transcript.

**Fig. 3:**
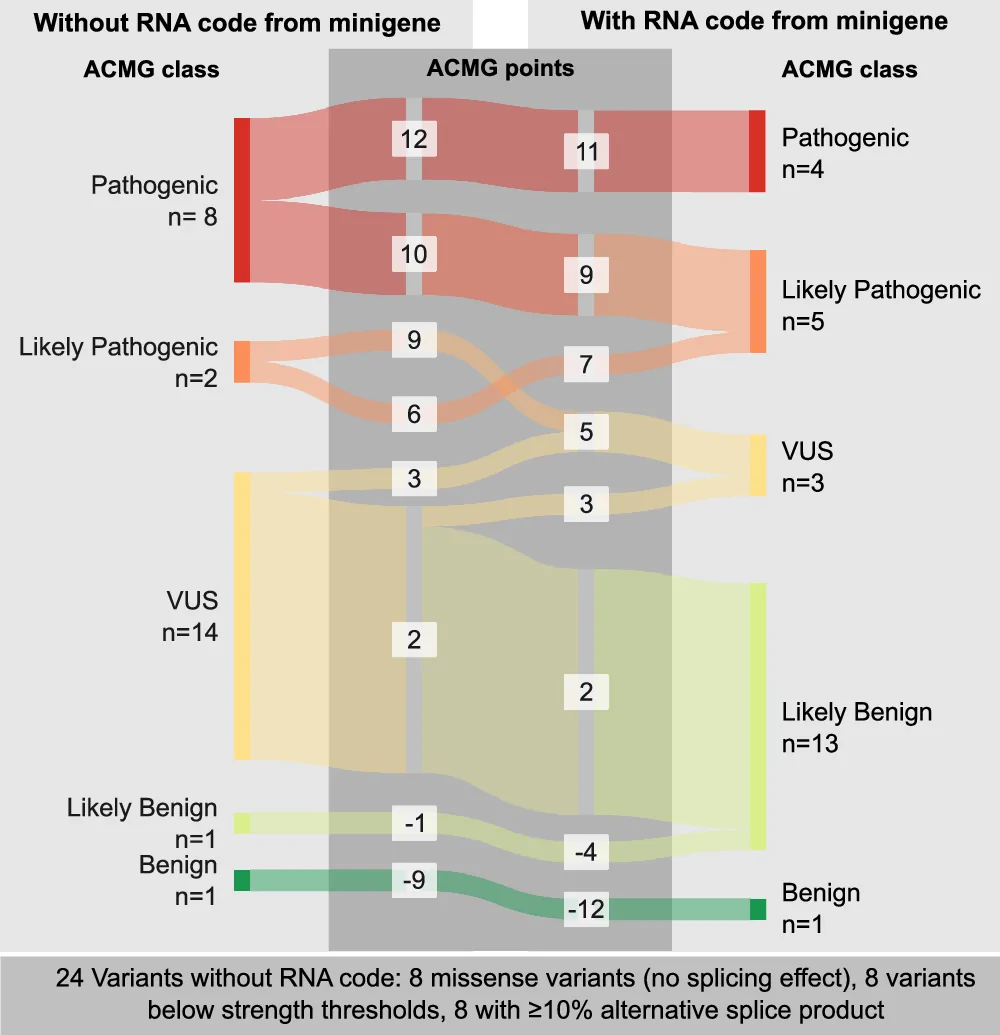
Overview of point- and class-level changes before and after integrating RNA evidence. (Diagram created using SankeyMATIC).

To assess the added value of minigene-derived RNA analyses and PVS1/BP7 (RNA) codes, we compared the point-based and total five-class ACMG/AMP assessment with and without RNA data for 26 variants that received an RNA code (Fig. 3, and Supplementary Data 4). Incorporation of RNA evidence resulted in an average ACMG point change (Δ) of 2.7 points (median Δ = 3.5 points; range: 1–4), with an increase for three variants (11.5%) and a decrease for 23 variants (88.5%) (Fig. 3). A class-switch based on minigene data was identified for 17 variants (65.4%), resulting in a clinically meaningful reclassification for 13 variants (50%): 12 (of 13, 92.3%) were reclassified from VUS to likely benign, and one variant (c.402T>A, of 13, 7.7%) from likely pathogenic to VUS.

### Selected variants and comparison of minigene to primary cancer data

**Fig. 4:**
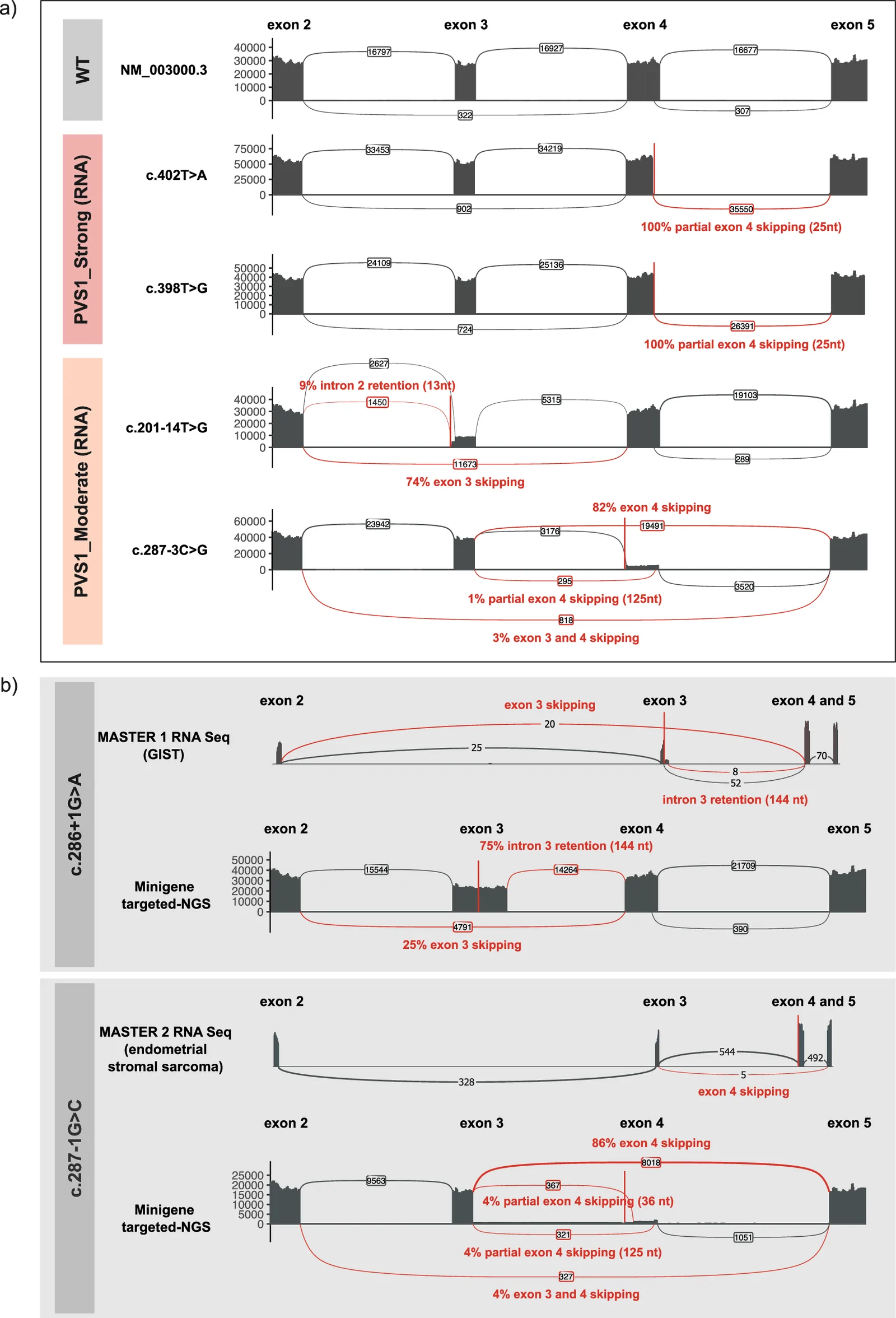
Splice effects for selected *SDHB* variants. **a** Sashimi plots of four selected variants showing aberrant splicing patterns in the minigene assay, with aberrant transcripts highlighted in red and variant positions indicated by red vertical lines. Only uniquely mapping reads were included. Cut off for splice junction reads in the visualization was 1% (of max. read count). ACMG/AMP RNA evidence codes were assigned based on minigene-derived transcript proportions as follows: c.402T*>*A (p.Tyr134Ter): 100% PVS1; c.398T*>*G (p.Met134Arg): 100% PVS1; c.201-14T*>*G (p.?): 83% PVS1 and 17% BP7_S (full-length wildtype); c.287-3C*>*G (p.?): 86% PVS1 and 14% BP7_S (full-length wildtype). **b** Sashimi plots of two splice-altering variants compared with tumor RNA sequencing data from two affected patients of the NCT/DKTK/DKFZ MASTER program. Aberrant transcripts are highlighted in red, and variant positions are indicated by red vertical lines. Minigene-derived transcript proportions were used to assign ACMG/AMP RNA evidence codes as follows: c.286+1G>A: 100% PVS1; c.287-1G>C: 93% PVS1, 4% PVS1_S and 4% N/A (intron 3 retention). GIST, gastrointestinal stromal tumor. (Created with ggsahimi).

We observed distinct aberrant and alternative splicing patterns for selected variants (Fig. 4a).

For two coding variants located in exon 4, aberrant splicing accounted for 100% of detected transcripts (Fig. 4a, top). Both the missense variant c.398T>G (p.Met133Arg) and interestingly, the stop-gain variant c.402T>A (p.Tyr134Ter), induced partial exon 4 skipping (r.399_423del, including the variant position c.402T>A) and resulted in frameshift transcripts with novel premature stop codons (p.(Met133ArgfsTer2) for c.398T>G and p.(Tyr134IlefsTer1) for c.402T>A). These findings revealed a splicing-based mechanism of pathogenicity, rather than the anticipated missense or direct truncating effect.

In addition to coding and splice-site variants, two intronic variants located outside canonical splice junctions exhibited pronounced splice disruption in the minigene assay (Fig. 4a, bottom). The intronic variant c.201-14T>G in the polypyrimidine tract resulted in extensive aberrant splicing, with more than 80% of transcripts harboring a premature termination codon due to exon 3 skipping (74%, r.201_286del, p.(Cys68HisfsTer21)) or a 13-nt intron 2 retention (9%, r.200_201ins[201-13_201-1], p.(Lys67AsnfsTer4)). The intronic variant c.287-3C>G predominantly induced exon 4 skipping (r.287_423del, p.(Ile97PhefsTer11)), accounting for 82% of all detected transcripts, with two additional low-abundance splice alterations including skipping of two exons (3 and 4; 3%) and a 125-nt partial exon 4 skipping (1%). Overall, 86% of aberrant transcripts were predicted to lead to premature termination codons. Furthermore, for eight variants, we detected the in-frame 54-nt partial exon 4 skipping event (r.370_423del, p.(Val124_Pro141del)), which was also observed in the wild-type minigene and normal tissues at very low levels (<1%), with an abundance ≥10% (Table 1, and Supplementary Data 3). Notably, the synonymous variant c.372C>A (p.Val124=) almost exclusively produced this isoform (97%). Similarly, two canonical splice-site variants (c.423+2T>G and c.423+1G>T) yielded this alternative transcript at substantial levels (51% and 27%), in addition to a frameshifting transcript (r.399_423del, p.(Tyr134IlefsTer1)) with 49% and 73% relative proportions.

To further confirm the validity of our minigene assay, we compared minigene results to splicing in primary human cancers and identified two variants as germline alterations in two participants of the precision oncology study NCT/DKTK/DKFZ MASTER (Horak et al.^22^; Jahn et al.^23^) (Fig. 4b). Within this program, DNA sequencing of fresh frozen tumor and blood as well as RNA sequencing of fresh frozen tumor tissue is performed. For the exon 3 canonical donor variant c.286+1G>A (Fig. 4b, top), germline heterozygosity was confirmed in blood DNA (variant allele frequency (VAF) 48%). Tumor DNA sequencing from a gastrointestinal stromal tumor (GIST) demonstrated near-complete loss of the wild-type allele (VAF 93%), consistent with loss of heterozygosity (LoH) as a second hit. Tumor RNA sequencing (tumor cell content 89%) revealed exon 3 skipping and activation of a cryptic donor site within intron 3, resulting in a 144-nt intron 3 retention (r.286_287ins[286+1_286+144]). A heterozygous germline coding variant within this patient in exon 4 also showed loss of heterozygosity in tumor DNA (VAF in blood 56%, tumor 96%). However, in tumor RNA, the exonic variant was only partially present (VAF ~ 39%), which likely indicated nonsense-mediated decay of the allele with the pathogenic germline variant, alongside expression of the wild-type transcript from the other allele in normal tissue. In line with these observations, the minigene assay reproduced both splice events, demonstrating 75% intron 3 retention and 25% exon 3 skipping.

In a second patient with the heterozygous exon 4 canonical acceptor variant c.287-1G>C (VAF 55%), we interrogated DNA and RNA from a metastatic endometrial stromal sarcoma (Fig. 4b, bottom). Tumor DNA showed an unchanged VAF (53%) and no evidence of a second hit at the *SDHB* locus. RNA sequencing (tumor cell content 87%) revealed exon 4 skipping (r.287_423del, p.(Ile97PhefsTer11)) with a very low splice junction read count. Notably, another germline variant in exon 1 demonstrated preserved heterozygosity in tumor DNA (VAF in blood 42%, tumor 51%) but near-complete monoallelic expression in tumor RNA (VAF 100%), indicating extensive allele-specific transcript degradation by nonsense-mediated decay. In line with this observation, the minigene assay revealed predominant exon 4 skipping (86%) and additional low-level transcripts predicted to introduce PTCs (exon 3 and exon 4 skipping, partial exon 4 skipping) for this variant. Together, these observations support the validity of the minigene assay and showcase its suitability to quantify transcripts leading to PTC. Such analyses cannot routinely be performed in tumor tissue, as fresh-frozen tumor material is not routinely available and aberrant transcripts may exhibit low expression due to nonsense-mediated mRNA decay (NMD).

## Discussion

We established a minigene encompassing a critical region of *SDHB* spanning exons 2–5, enabling systematic assessment of 48 splice-associated variants prioritized by SpliceAI. Targeted NGS-based cDNA sequencing revealed substantial transcript diversity, with 22 unique aberrant transcripts detected across all tested variants. Aberrant splicing exceeding 10% of the total transcript abundance was observed for almost half of the investigated variants of uncertain significance (16 of 34). Integration of these transcriptional findings into an RNA-based ACMG/AMP evidence framework resulted in the assignment of RNA codes to approximately half of the variants, underscoring both the sensitivity of transcript-level assays and the challenges of translating complex splicing patterns into robust variant-level conclusions.

SpliceAI^8^ is a benchmarked^24^ and widely used in silico tool to explore potential splice-altering effects. Although high SpliceAI scores had a high predictive value for splice disruption, our data indicate that intermediate to high unmasked prediction scores (range 0.44-0.92) - particularly for non-canonical intronic and exonic variants - frequently correspond to predominantly wild-type splicing as observed for 40% of examined variants. While masked prediction scores would have reduced the proportion of false positive predictions to 29%, these findings underscore the limited specificity of in silico prioritization and the need for transcript-based functional validation, especially for deep intronic variants^8^. The limited sensitivity of SpliceAI for deep intronic variants^8^ could potentially be improved by incorporating ClinVar-listed variants into the prioritization strategy. In addition, novel in silico prediction tools or their combination might be beneficial^25,26^.

Comparison of targeted NGS-based transcript profiling with conventional gel electrophoresis followed by Sanger sequencing^10,27^ revealed that approximately half of the transcripts detected by NGS would likely have escaped detection by conventional methods. While this precludes direct comparison with fragment analysis commonly used in minigene assays^13,28,29^, it highlights the substantially increased sensitivity of NGS-based approaches to resolve complex splice events. These findings align with recent methodological advances emphasizing the complexity of transcript structures generated in minigene assays and the need for refined analytical strategies, including dedicated transcript reconstruction tools^30^ and long-read sequencing approaches that enable full-length transcript resolution and phasing^31^.

While targeted transcript analysis was highly sensitive for detecting low-abundance transcripts, translating transcript effects into robust evidence strengths remained challenging. Although transcript-level PVS1/BP7_strength assignments were possible for ≈90% of detected transcripts, only a subset of variants (≈50%) met conservative cut-offs for combined-strength aggregation. Reasons were the potential impact of missense variants on protein level, transcript fractions below predefined thresholds, or the presence of alternative splicing patterns. The incorporation of transcript-level data into an ACMG/AMP framework resulted in shifts of ACMG points^32^ for 54% of all tested variants, predominantly reflecting a reduction in inferred pathogenicity. However, these shifts did not consistently translate into clinically actionable classification changes, as we applied a conservative interpretation of minigene data with a maximum evidence weight of strong for functional splicing assays in line with *BRCA1/2* guidelines^21^. Notably, for Lynch syndrome-associated genes, minigene-based validation of aberrant splicing observed in constitutional normal tissues is recommended^33^. This underscores that transcript-based evidence is best interpreted as a complementary evidence layer and integrated with additional functional, clinical, and population-based information. Additionally, incomplete aberrant splicing might be of clinical relevance, as it could influence penetrance in hereditary PPGL and may also be relevant in the context of biallelic disease^34^. Consistent with the limitations observed in our dataset, large-scale functional studies using saturation genome editing (SGE) have demonstrated that functional scores can resolve the majority of variants across clinically relevant genes such as *BRCA2, BAP1, DDX3X* and *VHL*^35–39^, but a non-negligible fraction remains intermediate or unresolved, emphasizing that no single assay can fully adjudicate all variants^36,37^.

In line with this, alternative splicing events illustrated the complexity of variant interpretation. An in-frame transcript isoform (r.370_423del, p.(Val124_Pro141del) (ENST00000485515.6)) was detected at low levels in the wild-type minigene but increased by over 10% abundance in eight variants. As the protein-level impact of this isoform remains undefined, these variants could not be assigned an RNA-based ACMG code. Structural annotation (UniProt, PDB) suggested that the resulting 18-aa-deletion affects the Fer2_3 domain and may alter interaction interfaces (chain A/C) and β-sheet architecture. Notably, the alternative 54-bp in-frame skipping event has been experimentally demonstrated in vivo for the splice-donor variant c.423+1G>A by RT-PCR analysis of patient-derived tumor RNA from two individuals with PPGL^40^. A distinct splice-donor variant at the same position, c.423+1G>T, has likewise been identified in a PPGL patient^41^. Consistent with these observations, our minigene assay showed that c.423+1G>T generated the identical in-frame skipping at a relative proportion of 27%, supporting the clinical relevance of this isoform while highlighting the need for protein-level functional validation. Recently, a protein-level functional assay for *SDHB* variants has been reported, which could provide complementary insights beyond transcript-based readouts, especially for missense variants without impact on splicing^34^. Aberrant splicing was also observed for the missense variant c.398T>G (p.Met133Arg) and the stop-gain variant c.402T>A (p.Tyr134Ter), emphasizing that splice-altering effects are not restricted to canonical splice-site changes but can occur across variant classes. Sensitive transcript-based assays are therefore essential to detect variant-specific splice outcomes and may provide a functional framework for evaluating splice-modulating strategies, including splice-switching antisense oligonucleotides, engineered snRNA-based approaches, and small-molecule splicing modifiers^42–44^. Together, these observations highlight that integrative interpretation across multiple functional layers will be required to fully resolve the molecular and clinical consequences of splice-associated variants, possibly enabling therapeutic approaches.

Although genome-editing approaches provide complementary advantages by interrogating variants within their native genomic context and enabling transcriptome-wide readouts^35,45^, they introduce additional experimental and analytical complexity, including confounding effects of gDNA abundance, NMD and cellular fitness. For deep intronic variants, genome-editing strategies based on homology-directed repair (HDR) face additional technical constraints, whereas emerging base- and prime-editing approaches may expand the scope of functional interrogation^46,47^. In contrast, minigene assays offer a splicing-focused and interpretable readout that allows direct variant attribution and quantification of observed effects, making them particularly well suited for experimental validation of splice predictions, despite limitations related to their artificial genomic context with potentially skewed or incomplete transcript complexity and reduced tissue-specific regulation^48^. Recently developed barcoded minigene approaches further enable pooled, NGS-based assessment of splicing events at increased throughput^49^ although such strategies remain constrained by assay design and transcript detection.

A shared limitation of genome-editing and minigene assays is the potential divergence of tissue-specific splicing from biologically relevant effects. However, endogenous RNA sequencing of HEK293T cells revealed predominant expression of the *SDHB* MANE transcript (NM_003000.3), consistent with low alternative splicing across GTEx tissues. Additionally, a substantial overlap between minigene-derived and tumor-derived splice products was observed for selected variants. Nevertheless, evaluation of selected variants in iPSC-based models (e.g., chromaffin cells, sympathetic neurons, neural-crest–derived cells) could further approximate native, tissue-specific splice regulation. Interpretation of patient-derived RNA sequencing remains challenging, particularly in the context of nonsense-mediated decay and loss of heterozygosity, which can obscure allele-specific splice effects and distort quantitative inference from steady-state RNA. Consistent with this limitation, tumor RNA sequencing for two variants confirmed variant-consistent aberrant splicing but did not permit reliable quantification of variant-induced effects. Tumor RNA interpretation could be improved by phasing through long-read sequencing. In contrast, the minigene assay enabled direct, allele-independent measurement of splice outcomes, providing a complementary and mechanistically interpretable functional readout.

By implementing an *SDHB* exon 2–5 minigene model in HEK293T cells, we provided a validated framework for the systematic assessment of splice-associated variants. Integration of targeted NGS, semi-automated HGVS annotation, and ACMG/AMP-based curation framework enables a scalable strategy for assessing splice-associated variants of uncertain significance and thereby narrowing the diagnostic gap in *SDHB*-related diseases. Our findings emphasize that accurate variant interpretation requires identification of precise molecular consequences rather than reliance on generic loss-of-function assumptions, with relevance for both germline and somatic variant assessment and future therapeutic considerations. We expect that multiplexed minigene-based approaches, combined with genome-editing and complementary functional assays, will advance characterization of splice-associated variants. Such strategies hold promise for dissecting context- and tissue-dependent effects of *SDHB* biology and for informing integrative models of variant interpretation and precision medicine.

## Methods

### Reference sequences and nomenclature

All genomic coordinates refer to the hg38/GRCh38 reference genome. Coding positions correspond to the *SDHB* MANE Select transcript (NM_003000.3/ENST00000375499.8), as defined by the Matched Annotation from NCBI and EMBL-EBI (MANE) project^50^ and amino acid positions and annotation of domains refer to the SDHB protein reference NP_002991.2 (*SDHB* gene – Gene ID: 6390)^51^ and UniProt P21912 (The UniProt Consortium)^52^. Variant nomenclature follows HGVS recommendations^53^. The plasmid sequence of the *SDHB* minigene and reference files are available on GitHub (GitHub/*SDHB*_minigene/04_Minigene_Reference). Additional variant annotations were sourced from Ensembl Variant Effect Predictor^54^.

Public transcript expression data from the Genotype-Tissue Expression (GTEx^18^) project were consulted to assess the tissue-wide expression of annotated *SDHB* transcript isoforms. Data were accessed via the GTEx Portal (https://gtexportal.org).

### Variant selection/Bioinformatic analysis

We selected the genomic region for experimental assessment of splice-associated *SDHB* variants based on functional considerations. Specifically, we included exons 2–5 and their flanking intronic sequences as they encompass essential protein domains and contain out-of-frame exon lengths. We systematically generated all possible single-nucleotide variants (SNVs) (GitHub/*SDHB*_minigene/01_Synthetic_Variant_Table) across the selected *SDHB* region and annotated them using the Ensembl Variant Effect Predictor (VEP; accessed 30.06.2023; GitHub/*SDHB*_minigene/03_Variant_Prioritization). Splice-associated variants were predicted with the in silico prediction tool SpliceAI using raw REF/ALT VCF mode, unmasked scores, and a 2000 bp prediction window (GitHub/*SDHB*_minigene/02_SpliceAI_Run)^8^. Unmasked scores were used for variant prioritization to sensitively capture potential aberrant splicing. We used a permissive maximum delta score ≥0.2 to maximize sensitivity for detecting potential splice-altering variants^24^. From the resulting dataset, 48 splice-associated variants were selected (GitHub/*SDHB*_minigene/03_Variant_Prioritization) for subsequent experimental assessment, ensuring representation of canonical splice sites as well as exonic and intronic positions. Masked scores were added retrospectively to facilitate interpretation of variants potentially affected by annotated alternative splicing events and to assess possible false-positive predictions.

### Minigene cloning and mutagenesis

The *SDHB* minigene was designed using the NEBuilder Assembly Tool and cloned into a linearized pcDNA3.1/Hygro(-) backbone (5596 nt) (GitHub/*SDHB*_minigene/ 04_Minigene_Reference) under the control of a CMV promoter (Addgene plasmid V875-20). Exons 2–5 together with their flanking intronic regions (A1-A3; 2905 nt. total length; GitHub/*SDHB*_minigene/04_Minigene_Reference) were amplified from 100 ng genomic DNA from HEK293T and human blood (Supplementary Fig. 1). Amplicon A1 (639 bp; chr1:17,044,436–17,045,090, GRCh38/hg38) comprised 202 bp of intron 1, exon 2 (128 bp), and 325 bp of intron 2 and was amplified from human blood genomic DNA. Amplicon A2 (759 bp; chr1:17,032,736–17,033,470) comprised 325 bp of intron 2, exon 3 (86 bp), and 325 bp of intron 3 and was amplified from HEK293T genomic DNA using the Chr1_MU27333v1_fix reference sequence to account for the hg38 fix at 1-17033181-G-T. Amplicon A3 (1,553 bp; chr1:17,027,547–17,029,061) comprised 325 bp of intron 3, exon 4 (137 bp), intron 4 (734 bp), exon 5 (117 bp), and 202 bp of intron 5 and was amplified from HEK293T genomic DNA using the Chr1_MU27333v1_fix reference sequence to account for the hg38 fix at 1-17028892-A-G. Amplicons were generated from 100 ng of template DNA in 50 µL reactions using Q5® High-Fidelity DNA Polymerase (NEB, M0491). Each reaction contained 1× Q5 Reaction Buffer, 1× Q5 High GC Enhancer, Q5 High-Fidelity DNA Polymerase (1U), 10 mM dNTPs (1 µl) and 2.5 µL of 10 µM *SDHB*-specific forward and reverse primers ( ≥ 20 nt; metabion; Supplementary Table 1). Primers included non-priming 5′ overhangs ( ≥ 25 bp) homologous to the 5′-terminal sequence of the adjacent amplicon and a gene specific 3′ region. PCR cycling conditions were: denaturation at 98 °C for 30 s; 30 cycles of 98 °C for 30 s, annealing at primer Tm (58–67 °C) for 45 s, and extension at 72 °C for 1 min; followed by final extension at 72 °C for 10 min. The vector backbone was digested with BamHI-HF / XhoI (NEB, R3136S / R0146S) at 37 °C for 1 h. Sticky-end fragments were assembled using the NEBuilder HiFi DNA Assembly Cloning Kit (M5520G) at a 2:1 insert-to-vector ratio and incubation of the reaction at 50 °C for 60 min. Assembled plasmids (8441 bp) were transformed into chemically competent *E. coli* DH5α cells (NEB, C29871) by heat shock (42 °C, 45 s), followed by recovery in LB medium for 30 min. at 37 °C and selection on ampicillin agar plates (100 µg/ml). Inserts were screened by colony PCR using primers: MG_Val_Fwd and MG_Val_Rev (Supplementary Table 1), followed by agarose gel electrophoresis (1% agarose, 1 h 15 min, expected amplicon size 3029 bp). Selected clones were purified using the plasmid Miniprep Kit (Qiagen, 27106) and minigene plasmid sequence was validated by next-generation sequencing (Illumina DNA Prep Flex; NextSeq 2000).

Variants were introduced into the minigene using site-directed mutagenesis, performed with a QuikChange-style protocol with modified primer design^55^. Reactions (20 µl) contained 30 ng of wildtype plasmid DNA, Q5® High-Fidelity DNA Polymerase (0.4 U), 1× High GC-Enhancer, 1× Q5 Reaction Buffer, 10 mM dNTPs (0.4 µl), and 1 µl each of 10 µM forward and reverse overlapping primers (Supplementary Table 1). Primers were designed in SnapGene (v6.0.3) with a total length of 50 bp, including 20 bp upstream and 29 bp downstream of the targeted SNV. PCR cycling conditions were 98 °C for 30 s; 30 cycles of 98 °C for 30 s, 72 °C for 45 s and 72 °C for 8 min; followed by 72 °C for 10 min. For variants located in A/T-rich regions that failed under standard protocol (c.424-151T>G, c.201-14T>G, c.232A>T), conditions were adapted^56^. PCR products were treated with DpnI (NEB, R0176L) at 37 °C for 1 h to remove methylated parental plasmid DNA and transformed into DH5α cells (NEB, C29871) as described above. Colonies were cultured with ampicillin (100 µg/ml) for 24 h at 37 °C and resistant colonies were screened by PCR and Sanger sequencing of the corresponding amplicon (Microsynth).

### Cell culture/Transfection

Wildtype and mutant minigenes were transfected separately into HEK293T cells maintained in DMEM/GlutaMAX medium (Gibco, 31966-021) supplemented with 10% FBS (Gibco, 10270106) in a humidified incubator at 37 °C and 5% CO_2_. Cell cultures were routinely tested for mycoplasma contamination and confirmed to be negative. As the minigene construct did not contain translational start or stop codons or untranslated regions (UTRs), nonsense-mediated decay (NMD) was not expected and therefore not inhibited. Cells were expanded to ~90% confluency in T75 flasks (~3 × 10^6^ cells) and 3.5 × 10⁵ cells were seeded into 12-well plates 24 h before transfection. Transfections were performed using 2.5 µl of Lipofectamine 3000 reagent (Invitrogen, L3000008), 5 µl of P3000 reagent (Invitrogen, L3000008), 100 µl Opti-MEM I (Gibco, 31985062), and 2.5 µg plasmid DNA per well. Twenty-four hours after transfection, cells were transferred to 6-well plates to increase RNA yield, using 1 ml PBS (Sigma-Aldrich, D5652-10X1L) and 0.5 ml trypsin solution (PAN-Biotech, P10-022100) per well. Cells were harvested 48 h post transfection using PBS, and RNAprotect Cell Reagent (Qiagen, 76526), yielding an average of ~1.77 × 10⁶ cells. RNA was stored at -80°C until further processing.

### RNA extraction and targeted RT-PCR

RNA was extracted from HEK293T cells using the RNeasy Mini Kit (Qiagen, 74104), QIAshredder (Qiagen, 79656) and RNase-Free DNase Set (Qiagen, 79256) according to the manufacturer’s instructions. RNA yield was quantified using the Qubit RNA BR Assay-Kit (Invitrogen, Q10210). For first strand cDNA synthesis (20 µl reaction volume), 1 µg RNA was mixed with 1 µl Oligo(dT)_12-18_ primer (Invitrogen, 18418012) and 1 µl of 10 mM dNTPs (NEB, N0447S) heated to 65 °C for 5 min and immediately chilled on ice. First Strand Buffer (1×, Invitrogen, Y02321), 0.1 M DTT (Invitrogen, PIN Y00147) and 1 µl RNasin Ribonuclease Inhibitor (Promega, N251A) were added. After incubation at 42 °C for 2 min, 1 µl SuperScript II (Invitrogen, 18064-022) was incorporated and cDNA synthesis proceeded at 42 °C for 1 h, followed by enzyme inactivation at 70 °C for 15 min. The resulting cDNA was treated with RNase H (Qiagen, Y9220L) at 37 °C for 20 min and 2 µl were used as template for PCR. PCR reactions (20 µl) contained Q5 High-Fidelity DNA Polymerase (0.2 µl), 1× High GC-Enhancer, 1× Q5 Reaction Buffer, 10 mM dNTPs (0.4 µl), and 1 µl each of 10 µM forward and reverse primers (RT_PCR_plasmid_fwd and RT_PCR_plasmid_rev; Supplementary Table 1). PCR amplification was performed under standardized cycling conditions (98 °C for 30 s; 30 cycles of 98 °C for 30 s, 65 °C for 45 s and 72 °C for 2 min; followed by 72 °C 10 min), selectively amplifying *SDHB* minigene-derived transcripts (wildtype-amplicon: 931 bp).

### Transcriptional analysis

#### Library preparation and sequencing

Targeted RT-PCR products were prepared for sequencing using the Illumina DNA Prep kit (20018704) according to the manufacturer’s protocol. Indexed libraries were sequenced on an Illumina NextSeq 2000 platform generating paired-end 2×150 bp reads, with a minimum depth of 50,000 reads per sample. Raw read quality was assessed with FastQC, and alignment quality metrics were obtained from STAR’s Log.final.out and SJ.out.tab files.

#### Read alignment and splice-junction quantification

Sequencing data were processed using the Spliced Transcripts Alignment to a Reference (STAR) algorithm^57^, adapted to a custom minigene reference and optimized parameter settings (GitHub/*SDHB*_minigene/05_NGS_Workflow). Alignment output consisted of BAM files and splice-junction files (SJ.out.tab) for downstream analyses.

#### Junction detection and splicing annotation

For each variant, all splice junctions contributing >1% of junctional reads relative to the most abundant wild-type junction were annotated manually with respect to splicing effect (e.g., exon skipping, alternative donor/acceptor usage, cryptic exon inclusion), splice-motif category and supporting read counts. Full intron retentions do not generate splice-junction signals and were therefore manually identified using Integrative Genomics Viewer (IGV v2.12.2)^58^. Sashimi plots (Fig. 4) were generated using a modified version of the ggsashimi code, adapted to apply the read-count threshold (>1% relative proportion) and to display uniquely mapped junction-spanning reads only (GitHub/*SDHB*_minigene/ 08_Additional_Plots_Code).

#### Transcript reconstruction and quantification

Distinct transcripts were inferred from splice-junction assignments. Transcript defining splice junctions (${j}_{x}$) were identified during splice-junction annotation. A junction was considered transcript-specific if it occurred exclusively in a single transcript and was absent from the full-length product and other aberrant transcripts. In case of pseudo-exon inclusion, two splice junctions could be considered as transcript-defining. At least one read had to span both junctions to distinguish true pseudo-exon inclusion from isolated donor or acceptor gain. If both confirming junctions were present but showed different read coverage, the junction with the lower read count was used to represent the transcript.

Transcript proportions were quantified as percent-spliced-in (PSI)^59^, adapted to STAR SJ output, by using uniquely mapped reads. PSI represents the proportion of inclusion-supporting reads compared to all reads that cover the corresponding splicing event. Following the identification of all relevant splice junctions for each variant (i), a reference splice junction (${j}_{r}$) was selected. This junction corresponded to the full-length transcript and was shared across all transcripts within one variant, thereby representing the total pool of inclusion- and exclusion-supporting reads. If multiple reference junctions were suitable, the junction with the highest number of uniquely mapping reads was chosen. The relative transcript ratio ($T{R}_{{jx}}$) was calculated by dividing the number of uniquely mapping reads crossing the defining splice junction $U{R}_{{jx}}$ by the number of uniquely mapping reads crossing the reference splice junction $U{R}_{{jr}}$:

1 $$T{R}_{{jx}}=\frac{U{R}_{{jx}}}{U{R}_{{jr}}}$$

If the sum of all transcript-defining junction counts did not equal the reference count $\left(\sum U{R}_{{jn}}\ne U{R}_{{jr}}\right)$, an internal normalization step was applied. For each transcript-defining junction, a normalized ratio ($R{N}_{{jn}}$) was calculated as:

2 $$R{N}_{{jx}}=\frac{U{R}_{{jx}}}{{\sum }_{n=1}^{i}U{R}_{{jn}}}$$

This adjustment ensured that all transcript ratios collectively summed up to 1.

${UR}$ uniquely mapping reads crossing a splice junction

$${TR}$$ transcript ratio

${RN}$ normalized transcript ratio

${j}_{x}$ transcript (x) defining splice junction

${j}_{r}$ reference splice junction

i total number of splice junctions defining the transcripts

If the proportion of transcript-defining reads relative to the reference $\left(\frac{\sum U{R}_{{jn}}}{U{R}_{{jr}}}\right)$ was <0.9, this indicated a potential whole-intron retention, which could not be detected by STAR algorithm as it produced no splice junctions. If a whole intron retention was confirmed in reference alignment (IGV), its proportion was estimated from read coverage across the retained intron to the coverage of the corresponding wild-type exon. If intron coverage was <1% of maximal exon coverage, missing reads were attributed to mapping imprecision and background noise, and ratios of transcripts were normalized to add up to 1. For whole intron retentions indicating >1% coverage, an estimated transcript ratio (${ET}{R}_{{jx}}$) was derived from coverage profiles and converted into an estimated read count (${EU}{R}_{{jx}}$) to integrate into TR normalization.

3 $${EU}{R}_{{jx}}={ET}{R}_{{jx}}\cdot U{R}_{{jr}}$$

These estimated read counts were then incorporated into the normalization pipeline described above.

PSI values and inferred transcript structures were aggregated in a curated annotation table (Supplementary Data 3).

#### HGVS-based transcript and protein annotation

The effects on transcript and predicted protein level were described using HGVS nomenclature^53^. For insertions (intron retention) and deletions (partial or complete exon skipping), an automated procedure was implemented to assign the respective HGVS annotations (GitHub/SDHB_minigene/06_Automated_HGVS_Nomenclature). The algorithm reconstructed r.() RNA variants from the flanking splice-junction coordinates and inferred the corresponding p.() protein consequences by translating the altered mRNA sequence^60^. Complex splicing effects - including pseudo-exons, multi-exon skipping, indels and double intron retentions - were annotated manually (Supplementary Table 2) according to HGVS guidelines, using the UCSC genome browser and the ORF finder tool^61^.

#### Gel-based transcript analysis and Sanger sequencing

In addition to NGS-based quantification of splicing, transcript analysis was performed using agarose gel electrophoresis and Sanger sequencing. RT-PCR products were separated on a 1% agarose gel at 110 V for 75 min and visualized using a Fusion FX Edge imaging system (Vilber). To improve resolution of samples with multiple bands, PCR products were re-run on a 0.8% agarose gel for approximately 2 h at 110 V. Visible bands were excised from the gel, purified using the QIAquick Gel Extraction Kit (Qiagen, 28704) and re-amplified by nested PCR (RT-PCR primers and conditions) prior to Sanger sequencing. Resulting FASTA files were analyzed using the CLC Workbench (v21.0.3).

#### RNA Sequencing

Endogenous *SDHB* transcripts of untransfected HEK293T cells were sequenced on the Illumina NextSeq2000 platform following TruSeq Stranded mRNA library prep (Illumina, 20020594) and processed using the alignment and splice-junction analysis described above. Tumor RNA sequencing (Illumina) of MASTER patient 1 and 2 was derived from fresh frozen tumors from the NCT/DKTK/DKFZ MASTER program^22,23^. Patients consented to banking of tumor and control tissue, molecular profiling of both samples, and clinical data collection (S-206/2011, Ethics Committee of the Medical Faculty of Heidelberg University). The study was conducted in adherence to the Declaration of Helsinki.

#### Integration of minigene transcriptional data into ACMG workflow

PVS1 was used to capture loss-of-function evidence and BP7 to classify variants without aberrant splicing impact, following the recommendations from the ClinGen SVI Splicing Subgroup^20^.

We applied a PVS1 strength to each aberrant transcript according to an adapted version (Supplementary Fig. 2a) of the published PVS1 decision tree^19^ to integrate critical protein regions overlapping with the minigene. The 2Fe–2S ferredoxin-type domain (Pfam Fer2_3, amino acids 40–133 in NP_002991.2) was defined as a critical functional domain (Supplementary Fig. 2b).

For variants showing both full-length wildtype and aberrant transcripts, we followed the recommended approach of assigning a PVS1 or BP7 strength to each individual transcript, pooling transcripts with the same evidence strength, and then applying an appropriate conservative overall PVS1 (RNA) or BP7 (RNA) strength that considers the relative contribution of each transcript to the overall expression^20^ (Supplementary Fig. 2c). Considering the in vitro setting and published gene-specific expert recommendations, e.g., of the ClinGen expert panel for *BRCA1/2*^21^, we established cautious thresholds for the overall strength and downweighted the PVS1 criterion to a maximum strength of strong (Supplementary Fig. 2d). The BP7 (RNA) code was assigned using a conservative cutoff of ≥90% of full-length wildtype transcript^19–21^. Following evaluation of minigene-derived splicing outcomes, variants were further classified using additional lines of evidence curated in Franklin, a clinical-grade genetic variant interpretation platform (https://franklin.genoox.com - Franklin by Genoox). ACMG/AMP criteria were applied to variants in accordance with the general guidelines^7^.

## Supplementary information

Supplementary Information

Supplementary Data 1

Supplementary Data 2

Supplementary Data 3

Supplementary Data 4

## Data Availability

Variants and corresponding minigene assay readouts were submitted to ClinVar as functional data (accession numbers SCV007665569 - SCV007665616). The data generated in this study are available via controlled access in the German Human Genome-Phenome Archive (GHGA, data.ghga.de) under the GHGA Accession https://data.ghga.de/study/GHGAS45216821504142. Further details, including the data access policy for the study, can be found there.

## Code availability

All related code for the *SDHB* minigene project as well as minigene reference is available from GitHub (https://github.com/AnniKoehler/SDHB_minigene/tree/main).
