# Supplementary material for: Minigene-based characterization and classification of splice-associated variants in succinate dehydrogenase B
Source: NPJ Precis Oncol. 2026 Sep 8;10:349. doi: 10.1038/s41698-026-01685-7 (PMC13554259; doi:10.1038/s41698-026-01685-7)
Supplement: Supplementary file 1 [file 41698_2026_1685_MOESM1_ESM.pdf]

Supplementary information

Supplementary Figures **2**
Supplementary Figure 1 2
Supplementary Figure 2 3
Supplementary Figure 3 4
Supplementary Figure 4 4
Supplementary Figure 5 4
Supplementary Figure 6 6
Supplementary Figure 7 7
Supplementary Figure 8 7

Supplementary Data **8**
Supplementary Data 1 8
Supplementary Data 2 8
Supplementary Data 3 8
Supplementary Data 4 8

Supplementary Tables **8**
Supplementary Table 1 8
Supplementary Table 2 12

Supplementary References **15**

Supplementary Figures

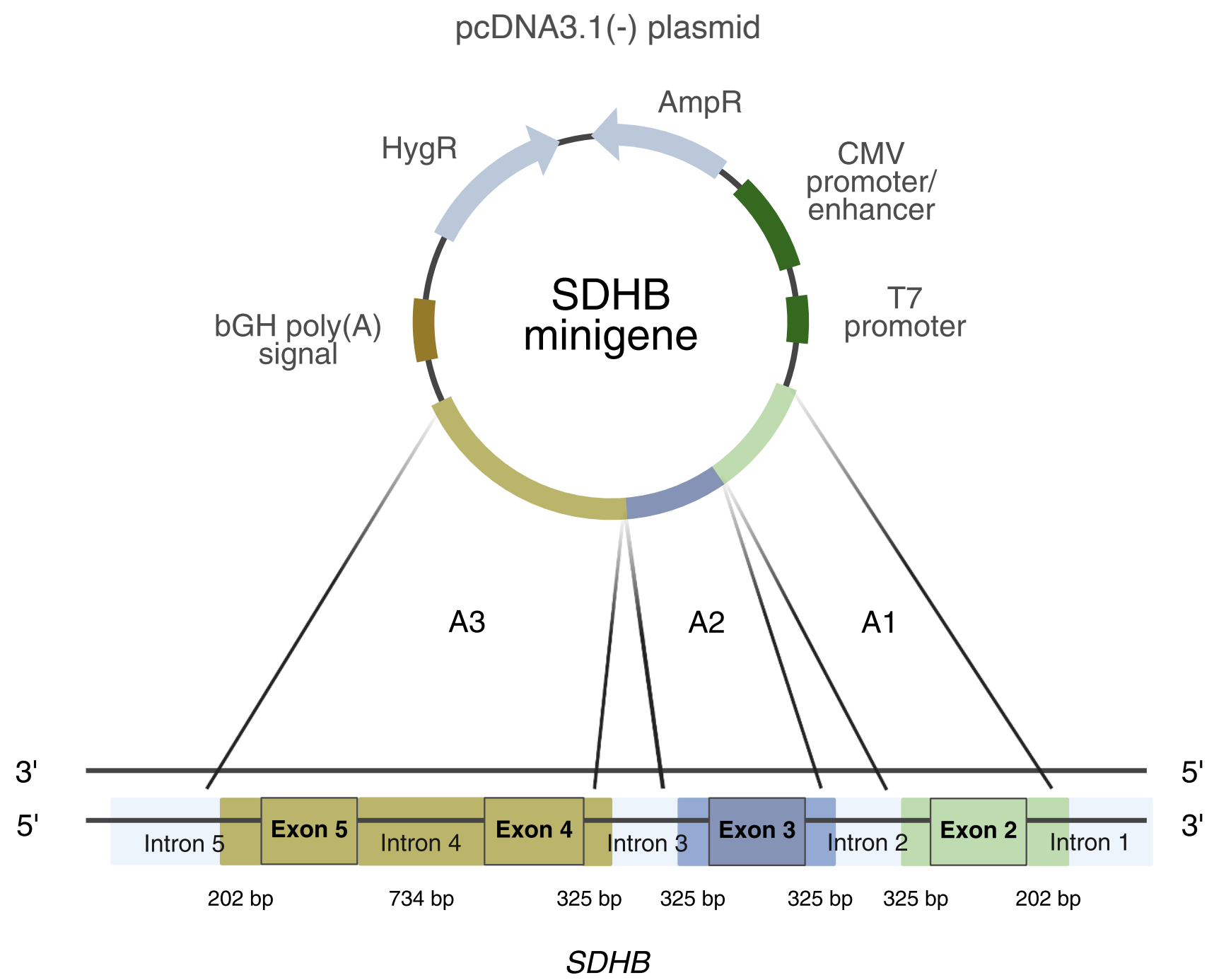

Supplementary Figure 1. Schematic representation of *SDHB* minigene with exon 2 to 5. (Created in BioRender. Köhler, A. (2026))

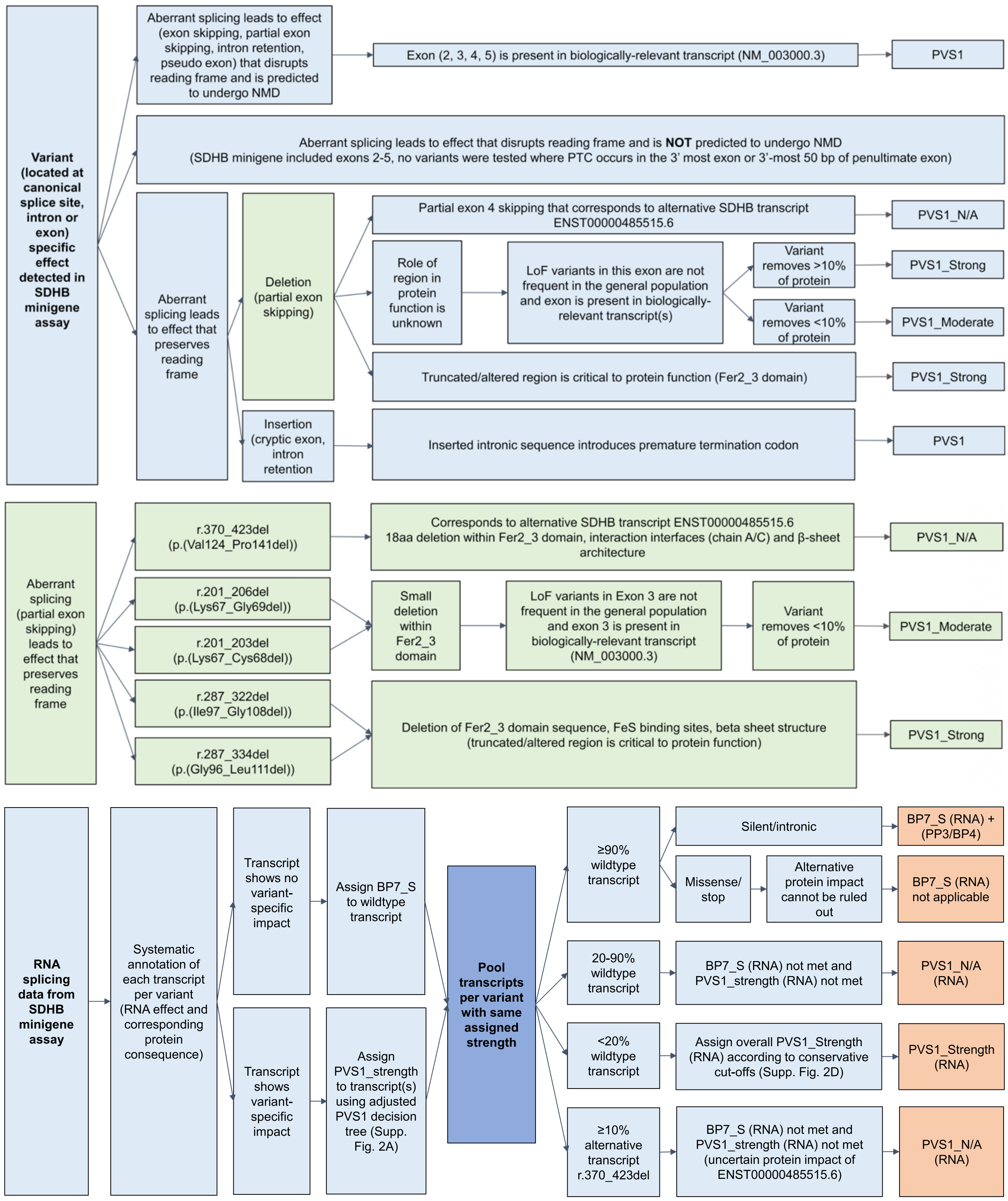

d)

Proportion of pooled aberrant transcripts produced by variant allele	Overall PVS1_Strength (RNA)
≥90% PVS1	PVS1_Strong (RNA)
≥90% PVS1_Strong or<br>80-90% PVS1	PVS1_Moderate (RNA)
≥90% PVS1_Moderate or<br>≥90% PVS1_Supporting or<br>80-90% PVS1_Strong / _Moderate / _Supporting	PVS1_Supporting (RNA)
**If multiple PVS1_strength categories are present within one variant, the overall strength is assigned according to the lowest category**	

Supplementary Figure 2. Decision frameworks for RNA-based ACMG/AMP interpretation of *SDHB* minigene assay results (Created in PowerPoint). a) PVS1 decision tree adapted from Abou Tayoun et al. [1] for interpretation of *SDHB* minigene assay results. b) PVS1 decision tree for in-frame transcripts observed in the *SDHB* minigene assay. c) Decision tree for overall ACMG/AMP classification based on RNA data derived from the *SDHB* minigene assay. d) Table summarizing conservative cut-offs for pooled aberrant transcripts, adapted from Parsons et al. [2].

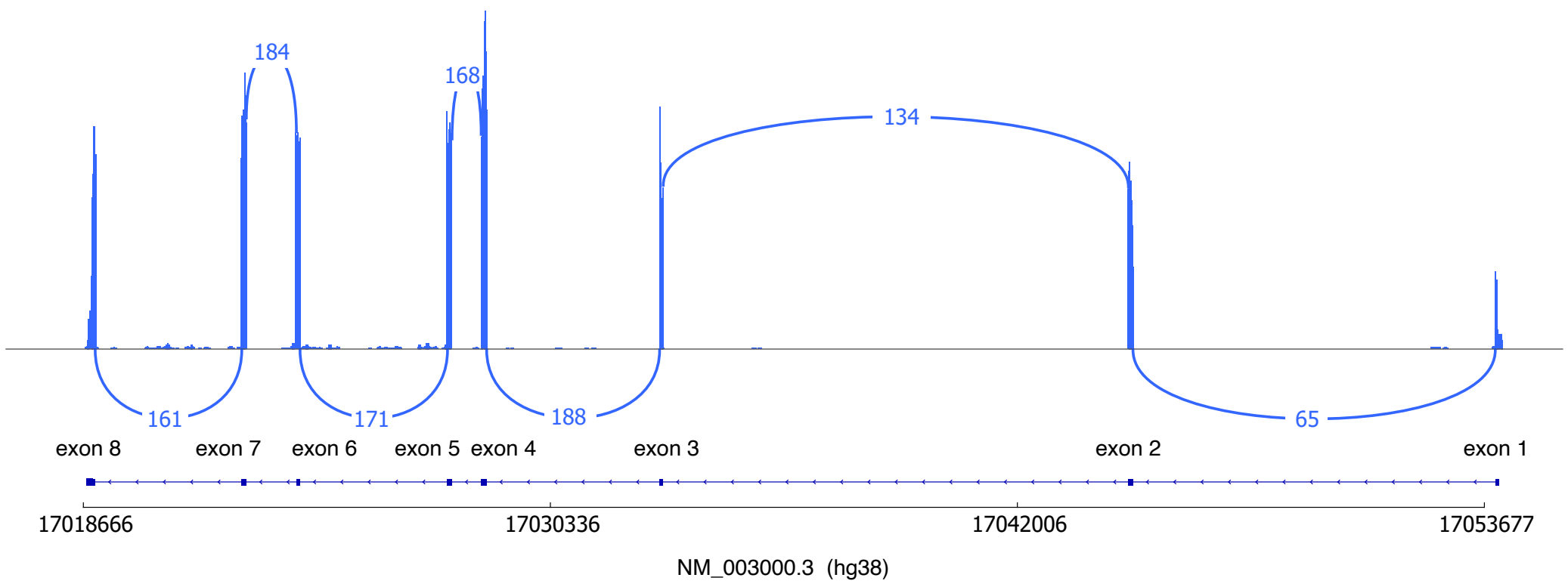

Supplementary Figure 3. RNA-Sequencing data of endogenous *SDHB* in HEK293T visualized as sashimi plot (Created in IGV).

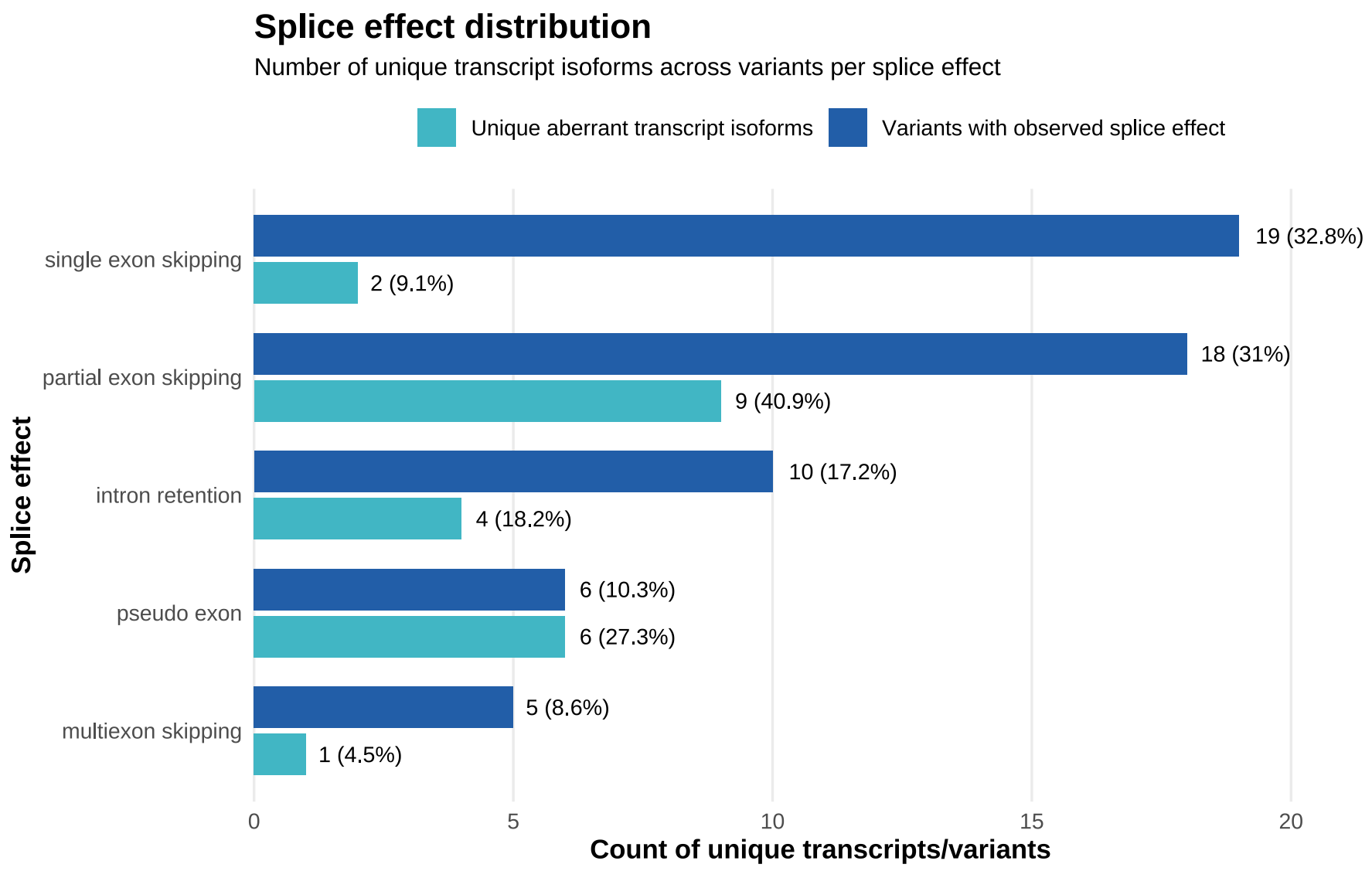

Supplementary Figure 4. Bar chart showing distribution of splice effects in unique transcript isoforms (n = 22) (created with R).

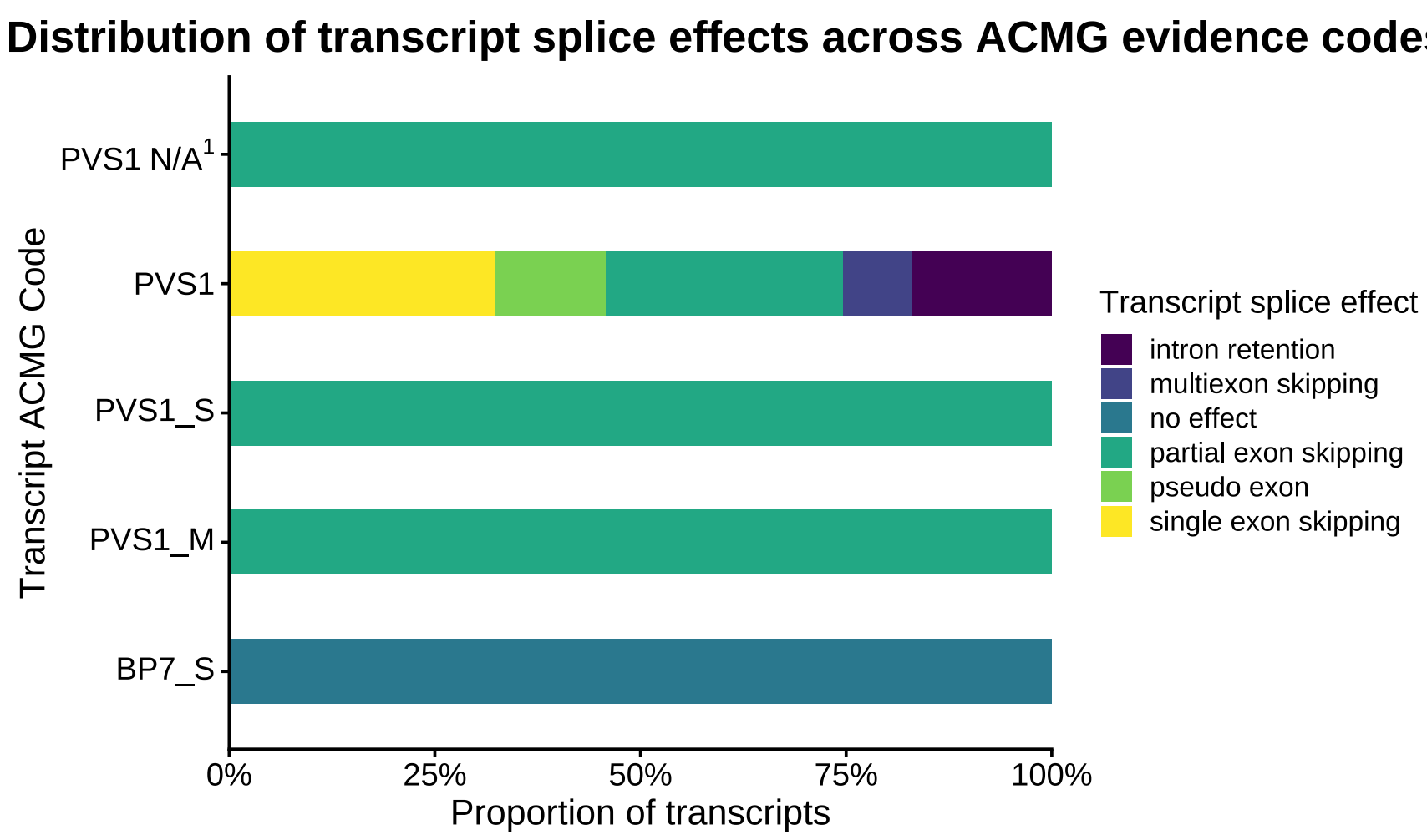

Supplementary Figure 5. Bar chart showing observed splice effects per assigned ACMG codes in all assessed transcripts (n=113). Footnotes: [1]Nine transcripts could not be assigned a strength code (PVS1_N/A), as all corresponded to an in-frame 54-nt partial exon 4 skipping (r.370_423del) matching the lowly expressed transcript ENST00000485515.6 (created with R).

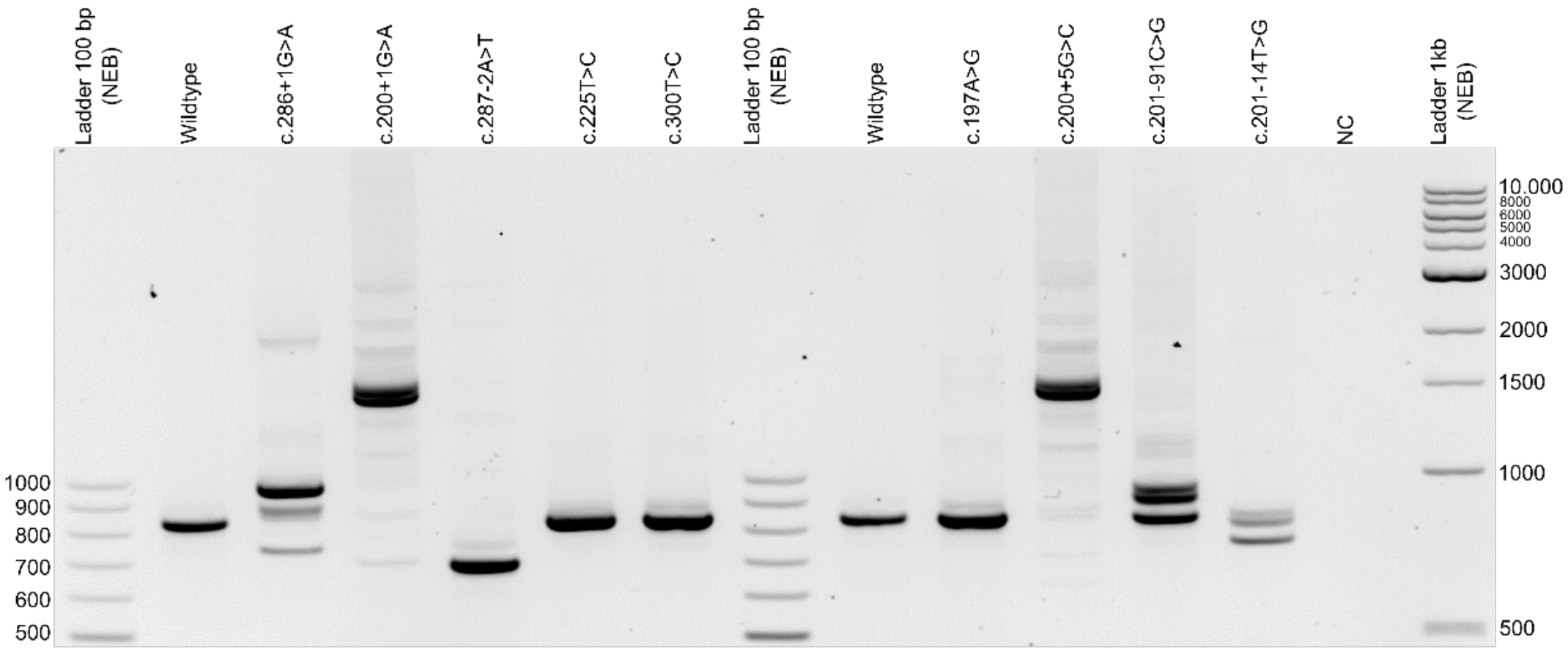
Ladder 100 bp (NEB)
Wildtype
c.286+1G>A
c.200+1G>A
c.287-2A>T
c.225T>C
c.300T>C
Ladder 100 bp (NEB)
Wildtype
c.197A>G
c.200+5G>C
c.201-91C>G
c.201-14T>G
NC
Ladder 1kb (NEB)
1000
900
800
700
600
500
10.000
8000
6000
5000
4000
3000
2000
1500
1000
500

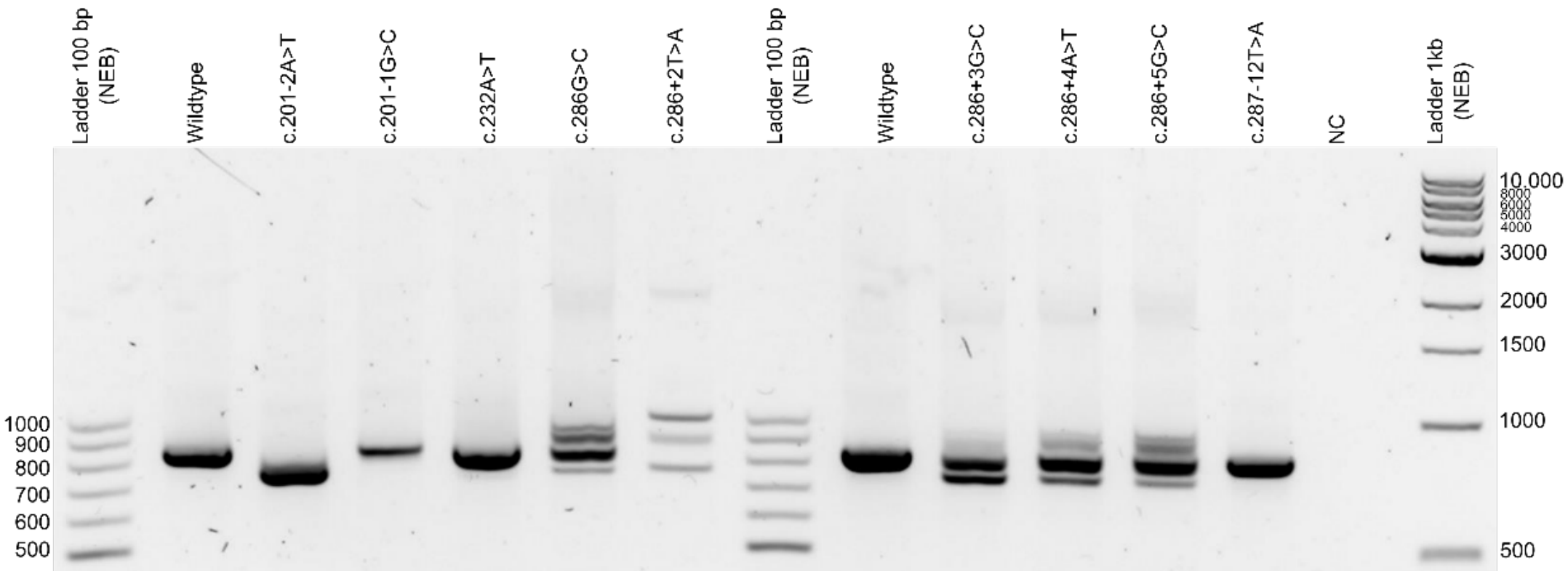
Ladder 100 bp (NEB)
Wildtype
c.201-2A>T
c.201-1G>C
c.232A>T
c.286G>C
c.286+2T>A
Ladder 100 bp (NEB)
Wildtype
c.286+3G>C
c.286+4A>T
c.286+5G>C
c.287-12T>A
NC
Ladder 1kb (NEB)
1000
900
800
700
600
500
10.000
8000
6000
5000
4000
3000
2000
1500
1000
500

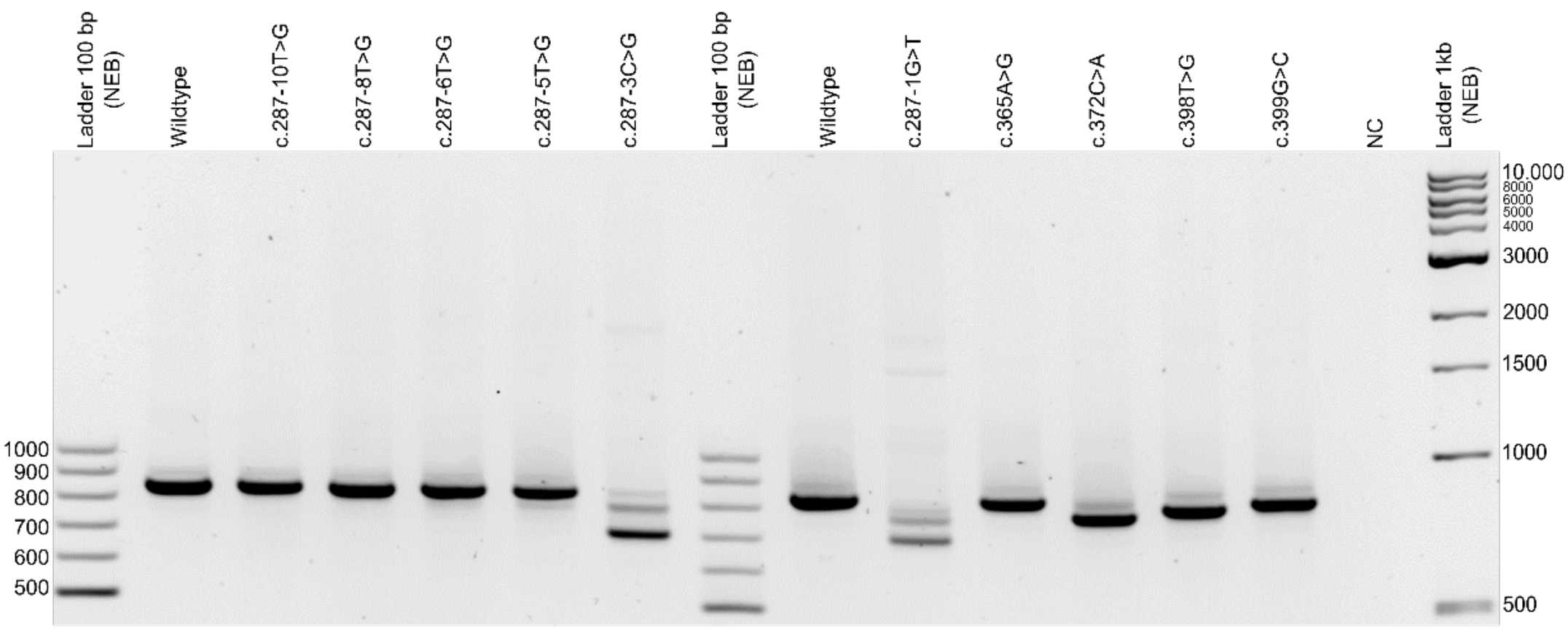
Ladder 100 bp (NEB)
Wildtype
c.287-10T>G
c.287-8T>G
c.287-6T>G
c.287-5T>G
c.287-3C>G
Ladder 100 bp (NEB)
Wildtype
c.287-1G>T
c.365A>G
c.372C>A
c.398T>G
c.399G>C
NC
Ladder 1kb (NEB)
1000
900
800
700
600
500
10.000
8000
6000
5000
4000
3000
2000
1500
1000
500

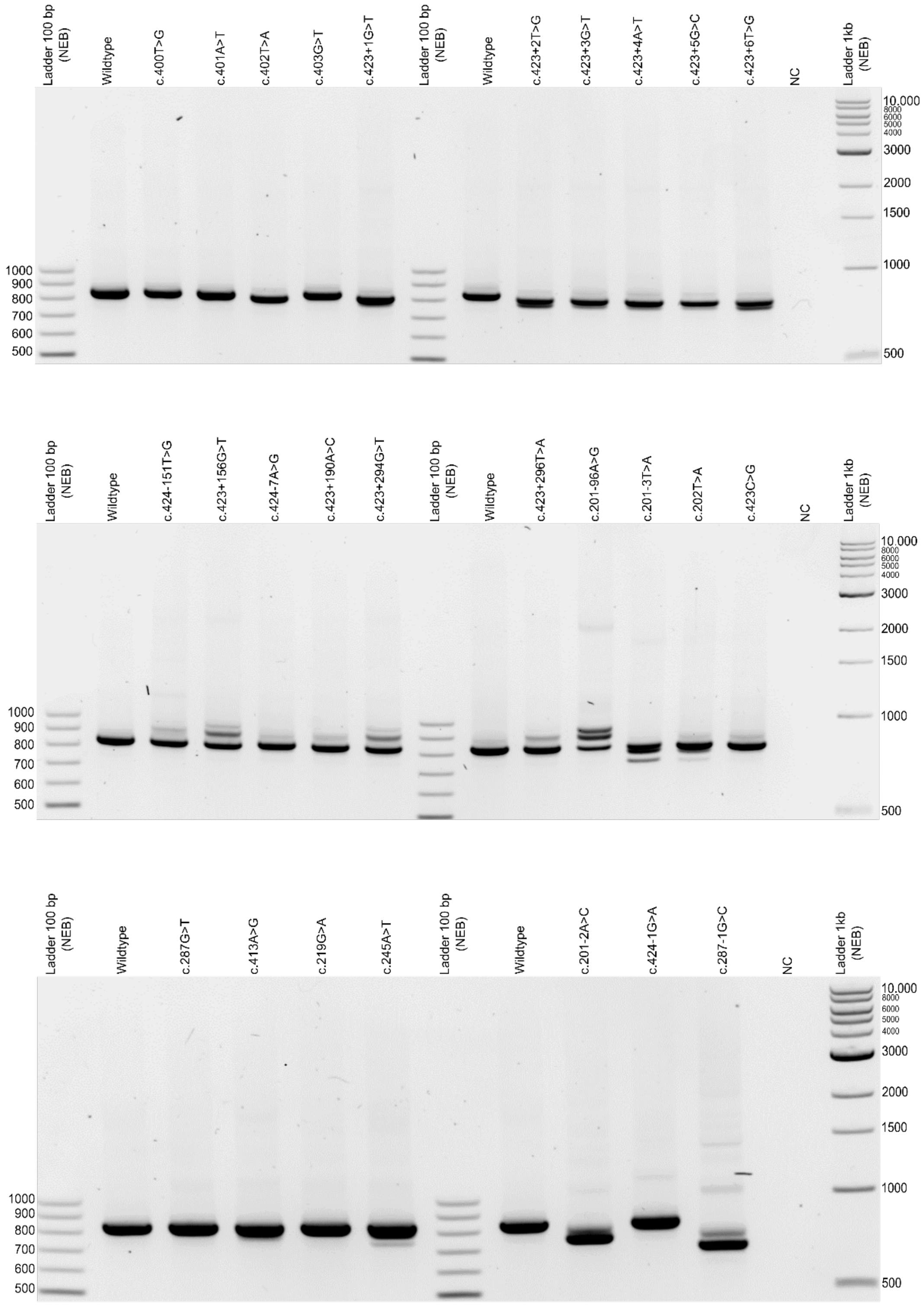

Supplementary Figure 6. Results from gel-electrophoresis of RT-PCR products, run at 110V for 1h 15min. in 1% agarose gel.

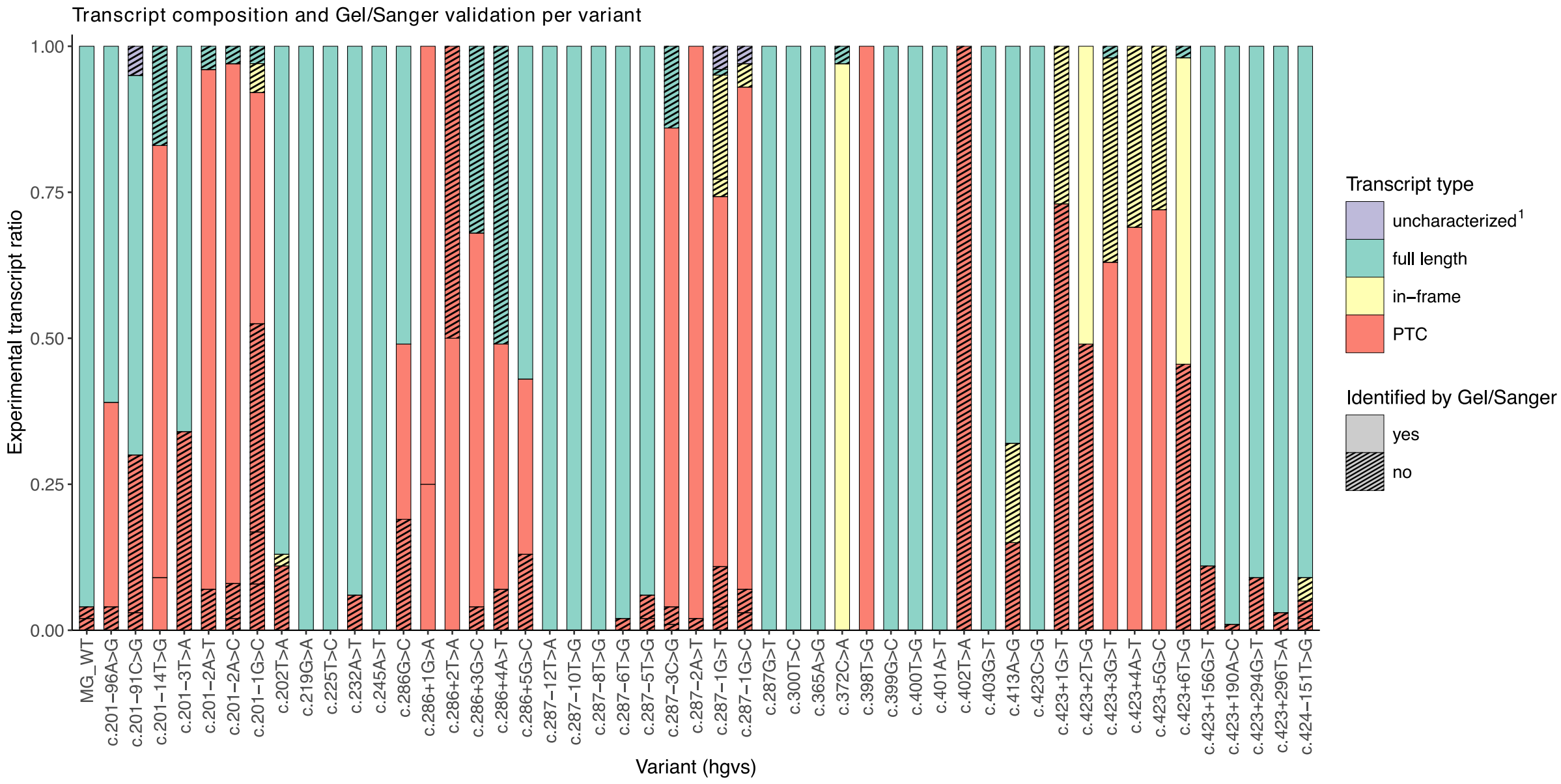

Supplementary Figure 7. Bar chart representing the distribution of transcript types observed in transcriptional analysis annotated with proportion of transcripts that could not be identified by Gel/Sanger method. Footnotes: [1]Low-abundance aberrant transcripts (<5%) corresponding to whole-intron retention events that could not be reliably interpreted in the endogenous genomic context because of the limited flanking intronic sequence included in the minigene construct (created with R).

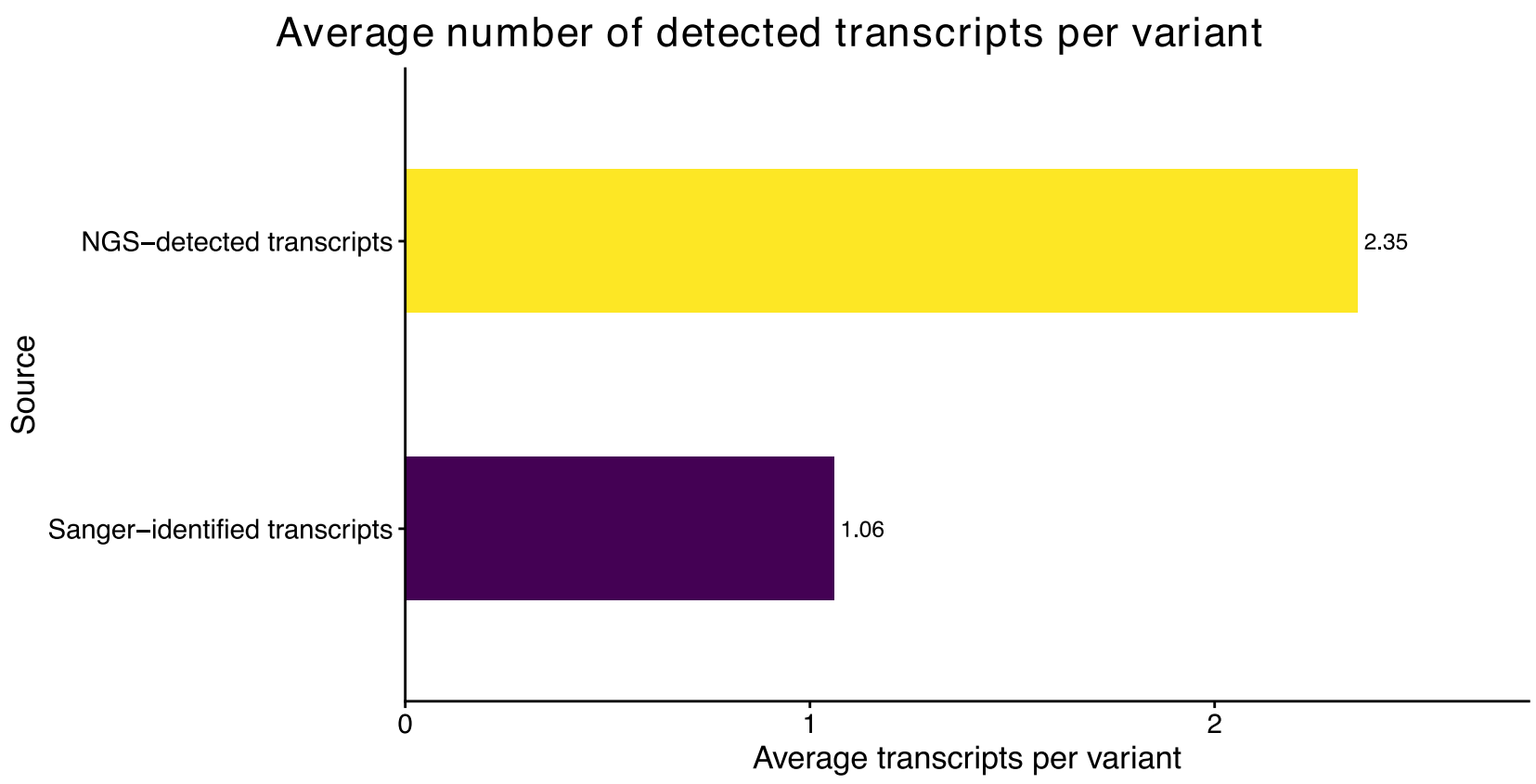

Supplementary Figure 8. Bar chart showing difference in resolution of transcript number comparing NGS approach with conventional Gel/sanger method (created with R).

Supplementary Data

Supplementary Data 1

SpliceAI prioritization of SDHB variants. Excel file containing SpliceAI-based prioritization results, including unmasked and masked delta scores and variant annotation from VEP.
File provided as “Supplementary Data 1.xlsx”.

Supplementary Data 2

Splice junction analysis of minigene-derived transcripts. Excel file containing splice-junction-level read counts and annotations derived from STAR SJ output for all analyzed variants and samples.
File provided as “Supplementary Data 2.xlsx”.

Supplementary Data 3

Transcript analysis results. Excel file containing curated transcript-level results, including inferred transcript structures, transcript ratios, and transcript classification across all assessed variants.
File provided as “Supplementary Data 3.xlsx”.

Supplementary Data 4

Extended data underlying Table 1. Excel file containing the extended version of Table 1, including detailed ACMG/AMP-based evidence assignments.
File provided as “Supplementary Data 4.xlsx“.

Supplementary Tables

Supplementary Table 1

Primer sequences used in this study. List of all primers used for SDHB minigene construction, site-directed mutagenesis, RT–PCR, and validation experiments. Primers were synthesized by metabion.

Name	T_m [°C]	Sequence 5'-3'	Use
Exon 2_HYG (-)_Fwd	63	tagcgtttaaacgggccctctagacGCCCCTAATACCATG TCTGATATATTAGCATATGATA	Amplicon PCR Minigene
Exon 2_rev	75	ttccaaataatttTGCCCCAGCCTCCTGAGTGG	Amplicon PCR Minigene
Exon 3_fwd	58	gaggctggggcaAAATTATTTGGAAAAACTTTGCAG	Amplicon PCR Minigene
Exon 3_rev	58	gttctagctccaCTTAATAAAAGAGCTCAGCAG	Amplicon PCR Minigene
Exon 4.5_fwd	67	ctcttttattaagTGGAGCTAGAACTGCTGTGG	Amplicon PCR Minigene
Exon 4.5_Rev HYG(-)	61	aaacttaagcttggtaccgagctcgCTCCTTGGTCCACTC TGGGC	Amplicon PCR Minigene
MG_Val_Fwd	58	TAATACGACTCACTATAGGGAGACCC	MG plasmid insert validation
MG Val Rev	58	CTGGCAACTAGAAGGCACAG	MG plasmid insert validation
RT_PCR_plasmid _fwd	58	AAGCTGGCTAGCGTTTAAACG	RT-PCR

RT_PCR_plasmid_rev	57	TAAACTTAAGCTTGGTACCGAGC	RT-PCR
MG_V1_Fwd	70	CCGAAGATCATGCAGAGAAGATGAGCATTTCATTCCTGTTGGGCTCCAGA	c.286+1G>A
MG_V1_Rev	67	CAACAGGAATGAAATGCTCATCTTCTCTGCATGATCTTCGGAAGGTCAAA	c.286+1G>A
MG_V2_Fwd	63	TATGAAGTTGACCTTAATAAATGAGTATCTCTGTGAAAGCCAGCTATTGA	c.200+1G>A
MG_V2_Rev	64	GGCTTTCACAGAGATACTCATTTATTAAGGTCAACTTCATAAGTCTGCAT	c.200+1G>A
MG_V3_Fwd	69	GTTAAATGTGTGTCTCTTTCGGGCATCTGTGGCTCTTGTGCAATGAACAT	c.287-2A>T
MG_V3_Rev	68	GCACAAGAGCCACAGATGCCCGAAAGAGACACACATTTAACACATCCTCA	c.287-2A>T
MG_V4_Fwd	65	GGCCCCATGGTATTGGATGCCTTAATCAAGATTAAGAATGAAGTTGACTC	c.225T>C
MG_V4_Rev	65	TCATTCTTAATCTTGATTAAGGCATCCAATACCATGGGGCCACATCTAAC	c.225T>C
MG_V5_Fwd	70	CTTTCAGGCATCTGTGGCTCCTGTGCAATGAACATCAATGGAGGCAACAC	c.300T>C
MG_V5_Rev	70	CCATTGATGTTCATTGCACAGGAGCCACAGATGCCTGAAAGAGACACACA	c.300T>C
MG_V7_Fwd	64	GACTTATGAAGTTGACCTTAGTAAGTGAGTATCTCTGTGAAAGCCAGCTA	c.197A>G
MG_V7_Rev	62	TTCACAGAGATACTCACTTACTAAGGTCAACTTCATAAGTCTGCATATGA	c.197A>G
MG_V8_Fwd	62	AAGTTGACCTTAATAAGTGACTATCTCTGTGAAAGCCAGCTATTGAAGGA	c.200+5G>C
MG_V8_Rev	65	AGCTGGCTTTCACAGAGATAGTCACTTATTAAGGTCAACTTCATAAGTCT	c.200+5G>C
MG_V9_Fwd	64	GAGAAGACCAAATGGATAAGGTAATACATCCAGGTGTCTCCGATTATATT	c.201-91C>G
MG_V9_Rev	66	GGAGACACCTGGATGTATTACCTTATCCATTTGGTCTTCTCAGGTCACCT	c.201-91C>G
MG_V10_Fwd	65	TACATAAATACCACTGGATAGTTTTCTTTGTTAGATGTGGCCCCATGGTA	c.201-14T>G
MG_V10_Rev	61	GCCACATCTAACAAAGAAAACTATCCAGTGGTATTTATGTAAAGTTCAAC	c.201-14T>G
MG_V11_Fwd	64	TTACATAAATACCACTGGATATTTTTCTTAGTTAGATGTGGCCCCATGGT	c.201-6T>A
MG_V11_Rev	64	ACCATGGGGCCACATCTAACTAAGAAAAATATCCAGTGGTATTTATGTAA	c.201-6T>A
MG_V12_Fwd	66	ACTGGATATTTTTCTTTGTTTGATGTGGCCCCATGGTATTGGATGCTTTA	c.201-2A>T
MG_V12_Rev	64	CAATACCATGGGGCCACATCAAACAAAGAAAAATATCCAGTGGTATTTAT	c.201-2A>T
MG_V13_Fwd	64	ACTGGATATTTTTCTTTGTTACATGTGGCCCCATGGTATTGGATGCTTTA	c.201-1G>C
MG_V13_Rev	65	CCAATACCATGGGGCCACATGTAACAAAGAAAAATATCCAGTGGTATTTA	c.201-1G>C
MG_V14_Fwd	62	TGGTATTGGATGCTTTAATCTAGATTAAGAATGAAGTTGACTCTACTTTG	c.232A>T
MG_V14_Rev	65	GTCAACTTCATTCTTAATCTAGATTAAAGCATCCAATACCATGGGGCCAC	c.232A>T
MG_V15_Fwd	69	TCCGAAGATCATGCAGAGAACGTGAGCATTTCATTCCTGTTGGGCTCCAG	c.286G>C
MG_V15_Rev	68	AACAGGAATGAAATGCTCACGTTCTCTGCATGATCTTCGGAAGGTCAAAG	c.286G>C
MG_V16_Fwd	69	CGAAGATCATGCAGAGAAGGAGAGCATTTCATTCCTGTTGGGCTCCAGAT	c.286+2T>A
MG_V16_Rev	68	CCAACAGGAATGAAATGCTCTCCTTCTCTGCATGATCTTCGGAAGGTCAA	c.286+2T>A
MG_V17_Fwd	67	GAAGATCATGCAGAGAAGGTCAGCATTTCATTCCTGTTGGGCTCCAGATA	c.286+3G>C
MG_V17_Rev	69	CCCAACAGGAATGAAATGCTGACCTTCTCTGCATGATCTTCGGAAGGTCA	c.286+3G>C

MG_V18_Fwd	69	AAGATCATGCAGAGAAGGTGTGCATTTCATTC CTGTTGGGCTCCAGATAC	c.286+4A>T
MG_V18_Rev	69	GCCCAACAGGAATGAAATGCACACCTTCTCTG CATGATCTTCGGAAGGTC	c.286+4A>T
MG_V19_Fwd	68	AGATCATGCAGAGAAGGTGACCATTTCATTCC TGTTGGGCTCCAGATACT	c.286+5G>C
MG_V19_Rev	71	AGCCCAACAGGAATGAAATGGTCACCTTCTCT GCATGATCTTCGGAAGGT	c.286+5G>C
MG_V20_Fwd	68	GTGAGGATGTGTTAAATGTGAGTCTCTTTCAG GCATCTGTGGCTCTTGTG	c.287-12T>A
MG_V20_Rev	67	CACAGATGCCTGAAAGAGACTCACATTTAACA CATCCTCACCCATATCCG	c.287-12T>A
MG_V21_Fwd	70	GAGGATGTGTTAAATGTGTGGCTCTTTCAGGC ATCTGTGGCTCTTGTGCA	c.287-10T>G
MG_V21_Rev	67	GCCACAGATGCCTGAAAGAGCCACACATTTAA CACATCCTCACCCATATC	c.287-10T>G
MG_V22_Fwd	69	GGATGTGTTAAATGTGTGTCGCTTTCAGGCAT CTGTGGCTCTTGTGCAAT	c.287-8T>G
MG_V22_Rev	68	GAGCCACAGATGCCTGAAAGCGACACACATTT AACACATCCTCACCCATA	c.287-8T>G
MG_V23_Fwd	68	ATGTGTTAAATGTGTGTCTCGTTCAGGCATCT GTGGCTCTTGTGCAATGA	c.287-6T>G
MG_V23_Rev	69	AAGAGCCACAGATGCCTGAACGAGACACACA TTTAACACATCCTCACCCA	c.287-6T>G
MG_V24_Fwd	68	TGTGTTAAATGTGTGTCTCTGTCAGGCATCTG TGGCTCTTGTGCAATGAA	c.287-5T>G
MG_V24_Rev	69	CAAGAGCCACAGATGCCTGACAGAGACACAC ATTTAACACATCCTCACCC	c.287-5T>G
MG_V25_Fwd	68	TGTTAAATGTGTGTCTCTTTGAGGCATCTGTG GCTCTTGTGCAATGAACA	c.287-3C>G
MG_V25_Rev	68	CACAAGAGCCACAGATGCCTCAAAGAGACAC ACATTTAACACATCCTCAC	c.287-3C>G
MG_V26_Fwd	66	TTAAATGTGTGTCTCTTTCATGCATCTGTGGCT CTTGTGCAATGAACATC	c.287-1G>T
MG_V26_Rev	69	TGCACAAGAGCCACAGATGCATGAAAGAGAC ACACATTTAACACATCCTC	c.287-1G>T
MG_V27_Fwd	67	AAGGATTGACACCAACCTCAGTAAGGTCTCAA AAATCTACCCTCTTCCAC	c.365A>G
MG_V27_Rev	67	GGTAGATTTTTGAGACCTTACTGAGGTTGGTG TCAATCCTTCGGGTGCAA	c.365A>G
MG_V28_Fwd	67	GACACCAACCTCAATAAGGTATCAAAAATCTA CCCTCTTCCACACATGTA	c.372C>A
MG_V28_Rev	66	GGAAGAGGGTAGATTTTTGATACCTTATTGAG GTTGGTGTCAATCCTTCG	c.372C>A
MG_V29_Fwd	67	AATCTACCCTCTTCCACACAGGTATGTGATAA AGGATCTTGTTCCCGTGA	c.398T>G
MG_V29_Rev	64	CAAGATCCTTTATCACATACCTGTGTGGAAGA GGGTAGATTTTTGAGACC	c.398T>G
MG_V30_Fwd	66	ATCTACCCTCTTCCACACATCTATGTGATAAAG GATCTTGTTCCCGTGAG	c.399G>C
MG_V30_Rev	64	ACAAGATCCTTTATCACATAGATGTGTGGAAG AGGGTAGATTTTTGAGAC	c.399G>C
MG_V31_Fwd	69	TCTACCCTCTTCCACACATGGATGTGATAAAG GATCTTGTTCCCGTGAGT	c.400T>G
MG_V31_Rev	63	AACAAGATCCTTTATCACATCCATGTGTGGAA GAGGGTAGATTTTTGAGA	c.400T>G
MG_V32_Fwd	68	CTACCCTCTTCCACACATGTTTGTGATAAAGG ATCTTGTTCCCGTGAGTT	c.401A>T
MG_V32_Rev	64	GAACAAGATCCTTTATCACAAACATGTGTGGA AGAGGGTAGATTTTTGAG	c.401A>T
MG_V33_Fwd	67	TACCCTCTTCCACACATGTAAGTGATAAAGGA TCTTGTTCCCGTGAGTTT	c.402T>A
MG_V33_Rev	63	GGAACAAGATCCTTTATCACTTACATGTGTGG AAGAGGGTAGATTTTTGA	c.402T>A
MG_V34_Fwd	67	ACCCTCTTCCACACATGTATTTGATAAAGGATC TTGTTCCCGTGAGTTTC	c.403G>T

MG_V34_Rev	64	GGGAACAAGATCCTTTATCAAATACATGTGTGGAAGAGGGTAGATTTTTG	c.403G>T
MG_V35_Fwd	66	TGATAAAGGATCTTGTTCCCTTGAGTTTCTGCATCTCTCTGGTTTTGTTT	c.423+1G>T
MG_V35_Rev	68	CAGAGAGATGCAGAAACTCAAGGGAACAAGATCCTTTATCACATACATGT	c.423+1G>T
MG_V36_Fwd	68	GATAAAGGATCTTGTTCCCGGGAGTTTCTGCATCTCTCTGGTTTTGTTTT	c.423+2T>G
MG_V36_Rev	66	CCAGAGAGATGCAGAAACTCCCGGGAACAAGATCCTTTATCACATACATG	c.423+2T>G
MG_V37_Fwd	65	ATAAAGGATCTTGTTCCCGTTAGTTTCTGCATCTCTCTGGTTTTGTTTTT	c.423+3G>T
MG_V37_Rev	67	ACCAGAGAGATGCAGAAACTAACGGGAACAAGATCCTTTATCACATACAT	c.423+3G>T
MG_V38_Fwd	66	TAAAGGATCTTGTTCCCGTGTGTTTCTGCATCTCTCTGGTTTTGTTTTTA	c.423+4A>T
MG_V38_Rev	67	AACCAGAGAGATGCAGAAACACACGGGAACAAGATCCTTTATCACATACA	c.423+4A>T
MG_V39_Fwd	64	AAAGGATCTTGTTCCCGTGACTTTCTGCATCTCTCTGGTTTTGTTTTTAT	c.423+5G>C
MG_V39_Rev	67	AAACCAGAGAGATGCAGAAAGTCACGGGAACAAGATCCTTTATCACATAC	c.423+5G>C
MG_V40_Fwd	68	AAGGATCTTGTTCCCGTGAGGTTCTGCATCTCTCTGGTTTTGTTTTTATT	c.423+6T>G
MG_V40_Rev	65	AAAACCAGAGAGATGCAGAACCTCACGGGAACAAGATCCTTTATCACATA	c.423+6T>G
MG_V41_Fwd	65	TTCCTTTTGTAATCTTTGCAGATATTCATGACAAAGGAAAAGAGTCTGGC	c.424-151T>G
MG_V41_Rev	62	TTTTCCTTTGTCATGAATATCTGCAAAGATTACAAAAGGAAAAATGGTCAC	c.424-151T>G
MG_V42_Fwd	68	GGGACTGTTTCCTATATTATTGCAGGAGGAGGACCACAGTGTCACTGAGA	c.423+156G>T
MG_V42_Rev	64	CACTGTGGTCCTCCTCCTGCAATAATATAGGAAACAGTCCCATCTATTTA	c.423+156G>T
MG_V43_Fwd	66	TCTTCTTCTTCTTCCTCTTAGCCACAGGATTTGAGCAACTTCTATGCACA	c.424-7A>G
MG_V43_Rev	65	AAGTTGCTCAAATCCTGTGGCTAAGAGGAAGAAGAAGAAGAAGAAGAAGA	c.424-7A>G
MG_V44_Fwd	67	CACAGTGTCACTGAGAGATTCGGAAAATCTCCAGATCTGAGACAGTGTGG	c.423+190A>C
MG_V44_Rev	68	CTCAGATCTGGAGATTTTCCGAATCTCTCAGTGACACTGTGGTCCTCCTC	c.423+190A>C
MG_V45_Fwd	68	CCAGCAGCTCATTTGGTGAGTTTTGTTTGAGAATAATGGGGCACAGAATT	c.423+294G>T
MG_V45_Rev	68	CCCCATTATTCTCAAACAAAACTCACCAAATGAGCTGCTGGAGGATTTTC	c.423+294G>T
MG_V46_Fwd	68	AGCAGCTCATTTGGTGAGGTATGTTTGAGAATAATGGGGCACAGAATTTT	c.423+296T>A
MG_V46_Rev	69	TGCCCCATTATTCTCAAACATACCTCACCAAATGAGCTGCTGGAGGATTT	c.423+296T>A
MG_V47_Fwd	68	GACCTGAGAAGACCAAATGGGTAAGCTAATACATCCAGGTGTCTCCGATT	c.201-96A>G
MG_V47_Rev	68	CACCTGGATGTATTAGCTTACCCATTTGGTCTTCTCAGGTCACCTTCGGA	c.201-96A>G
MG_V48_Fwd	67	CACTGGATATTTTTTCTTTGTAAGATGTGGCCCCATGGTATTGGATGCTTT	c.201-3T>A
MG_V48_Rev	64	AATACCATGGGGCCACATCTTACAAAGAAAAATATCCAGTGGTATTTATG	c.201-3T>A
MG_V49_Fwd	65	GGATATTTTTCTTTGTTAGAAGTGGCCCCATGGTATTGGATGCTTTAATC	c.202T>A
MG_V49_Rev	65	ATCCAATACCATGGGGCCACTTCTAACAAAGAAAAATATCCAGTGGTATT	c.202T>A
MG_V50_Fwd	66	GTGATAAAGGATCTTGTTCCGGTGAGTTTCTGCATCTCTCTGGTTTTGTT	c.423C>G
MG_V50_Rev	64	AGAGAGATGCAGAAACTCACCGGAACAAGATCCTTTATCACATACATGTG	c.423C>G

MG_V51_Fwd	70	ATGTGTGTCTCTTTCAGGCAGCTGTGGCTCTT GTGCAATGAACATCAATG	c.290T>G
MG_V51_Rev	68	TCATTGCACAAGAGCCACAGCTGCCTGAAAGA GACACACATTTAACACAT	c.290T>G
MG_V52_Fwd	66	TAAATGTGTGTCTCTTTCAGTCATCTGTGGCTC TTGTGCAATGAACATCA	c.287G>T
MG_V52_Rev	69	TTGCACAAGAGCCACAGATGACTGAAAGAGAC ACACATTTAACACATCCT	c.287G>T
MG_V53_Fwd	67	ACACATGTATGTGATAAAGGGTCTTGTTCCCG TGAGTTTCTGCATCTCTC	c.413A>G
MG_V53_Rev	68	AGAAACTCACGGGAACAAGACCCTTTATCACA TACATGTGTGGAAGAGGG	c.413A>G
MG_V54_Fwd	65	AGATGTGGCCCCATGGTATTAGATGCTTTAAT CAAGATTAAGAATGAAGT	c.219G>A
MG_V54_Rev	64	TTAATCTTGATTAAAGCATCTAATACCATGGGG CCACATCTAACAAAGAA	c.219G>A
MG_V55_Fwd	62	TTAATCTTGATTAAAGCATCTAATACCATGGGG CCACATCTAACAAAGAA	c.245A>T
MG_V55_Rev	64	AGGTCAAAGTAGAGTCAACTACATTCTTAATCT TGATTAAAGCATCCAAT	c.245A>T
MG_V56_Fwd	65	ACTGGATATTTTTCTTTGTTCGATGTGGCCCCA TGGTATTGGATGCTTTA	c.201-2A>C
MG_V56_Rev	64	CAATACCATGGGGCCACATCGAACAAAGAAAA ATATCCAGTGGTATTTAT	c.201-2A>C
MG_V57_Fwd	65	TCTTCTTCCTCTTAACCACAAGATTTGAGCAAC TTCTATGCACAGTACAA	c.424-1G>A
MG_V57_Rev	65	GCATAGAAGTTGCTCAAATCTTGTGGTTAAGA GGAAGAAGAAGAAGAAGA	c.424-1G>A
MG_V58_Fwd	66	TTAAATGTGTGTCTCTTTCACGCATCTGTGGCT CTTGTGCAATGAACATC	c.287-1G>C
MG_V58_Rev	69	TGCACAAGAGCCACAGATGCGTGAAAGAGAC ACACATTTAACACATCCTC	c.287-1G>C

Supplementary Table 2

HGVS nomenclature of transcripts with pseudo-exon inclusion, indels, double insertions, or multi-exon skipping. This table contains HGVS-based transcript annotations for aberrant transcripts involving pseudo-exon inclusion, insertions/deletions, double insertions, or multi-exon skipping. Open reading frames (ORFs) were identified using the ORF Finder tool (https://www.bioinformatics.org/sms2/orf_find.html). AA indicates the first altered amino acid. If the upstream exon ended with an incomplete base triplet (B or BB), these bases were placed upstream of the pseudo-exon sequence and included in the ORF analysis.

**Variant (HGVSc)**	RNA HGVS (effect)	Inserted/deleted sequence	Orf finder output	Protein HGVS (NP_002991.2)
**c.201-91C>G**<br><br>**Transcript 9b**	r.200-201ins[201-211_201-92]	Exon 2 end: AA (Lys 67)<br><br>AAATGTGTTTGCACTGTGATCATCAAAATAGAACTGTGCAGTACTTAAAATGTAAGAATCTGCAAAAACAAAACTACAGAACAAATTCCGAAGGTGACCTGAGAAGACCAAATGGATAAG<br><br>Exon 3 beginning: A TGT (Cys 68)	>ORF number 1 in reading frame 1 on the direct strand extends from base 1 to base 21.<br>AAAAATGTGTTTGCACTGTGA<br><br>>Translation of ORF number 1 in reading frame 1 on the direct strand.<br><br>KNVFAL*	Ter after 6 AA<br><br>p.Cys68AsnTer6
**c.201-91C>G**<br><br>**Transcript 9d**	r.200-201ins[201-226_201-92]	Exon 2 end: AA<br><br>CTTTCCATTTCCTAGAAATGTGTTTGCACTGTGATCATCAAAATAGAACTGTGCAGTACTTAAAATGTAAGAATCTGCAAAAACAAAACTACAGAACAAATTCCGAAGGTGACCTGAGAAGACCAAATGGATAAG	>ORF number 1 in reading frame 1 on the direct strand extends from base 1 to base 36.<br>AACTTTCCATTTCCTAGAAATGTGTTTGCACTGTGA	Ter after 12 AA<br><br>p.Lys67AsnTer12

		Exon 3 beginning: A TGT (Cys 68)	>Translation of ORF number 1 in reading frame 1 on the direct strand.<br><br>NFPFPRNVFAL*	
**c.201-96A>G**<br><br>**Transcript 47b**	r.200-201ins[201-211_201-97]	Exon 2 end: AA (Lys 67)<br><br>AAATGTGTTTGCACTGTGATCATCAAAATAGAACTGTGCAGTACTTAAAATGTAAGAATCTGCAAAAACAAAACTACAGAACAAATTCCGAAGGTGACCTGAGAAGACCAAATGG<br><br>Exon 3 beginning: A TGT (Cys 68)	>ORF number 1 in reading frame 1 on the direct strand extends from base 1 to base 21.<br>AAAAATGTGTTTGCACTGTGA<br><br>>Translation of ORF number 1 in reading frame 1 on the direct strand.<br><br>KNVFAL*	Ter after 6 AA<br><br>p.Cys68AsnTer6
**c.201-96A>G**<br><br>**Transcript 47c**	r.200-201ins[201-226_201-97]	Exon 2 end: AA (Lys 67)<br><br>CTTTCCATTTCCTAGAAATGTGTTTGCACTGTGATCATCAAAATAGAACTGTGCAGTACTTAAAATGTAAGAATCTGCAAAAACAAAACTACAGAACAAATTCCGAAGGTGACCTGAGAAGACCAAATGG<br><br>Exon 3 beginning: A TGT (Cys 68)	>ORF number 1 in reading frame 1 on the direct strand extends from base 1 to base 36.<br>AACTTTCCATTTCCTAGAAATGTGTTTGCACTGTGA<br><br>>Translation of ORF number 1 in reading frame 1 on the direct strand.<br><br>NFPFPRNVFAL*	Ter after 12 AA<br><br>p.Lys67AsnTer12
**c.423+156G>T**<br><br>**Transcript 42b**	r.423_424ins[423+161_423+288]	Exon 4 end: CCC (Pro 141)<br><br>GAGGAGGACCACAGTGTCACTGAGAGATTAGGAAAATCTCCAGATCTGAGACAGTGTGGCCAGAGTTTAATTAAAGAATTTGAATGGCAGAAGATAAACGCCTAGAAAATCCTCCAGCAGCTCATTTG<br><br>Exon 5 beginning: GAT (Asp 142)	>ORF number 1 in reading frame 1 on the direct strand extends from base 1 to base 105.<br>GAGGAGGACCACAGTGTCACTGAGAGATTAGGAAAATCTCCAGATCTGAGACAGTGTGGC<br>CAGAGTTTAATTAAAGAATTTGAATGGCAGAAGATAAACGCCTAG<br><br>>Translation of ORF number 1 in reading frame 1 on the direct strand.<br><br>EEDHSVTERLGKSPDLRQCGQSLIKEFEWQKINA*	Ter after 35 AA<br><br>p.Asp142GluTer35
**c.423+190A>C**<br><br>**Transcript 44b**	r.423_424ins[423+161_423+288]	Exon 4 end: CCC (Pro 141)<br><br>GAGGAGGACCACAGTGTCACTGAGAGATTCGGAAAATCTCCAGATCTGAGACAGTGTGGCCAGAGTTTAATTAAAGAATTTGAATGGCAGAAGATAAACGCCTAGAAAATCCTCCAGCAGCTCATTTG<br><br>Exon 5 beginning: GAT (Asp 142)	>ORF number 1 in reading frame 1 on the direct strand extends from base 1 to base 105.<br><br>GAGGAGGACCACAGTGTCACTGAGAGATTCGGAAAATCTCCAGATCTGAGACAGTGTGGC<br>CAGAGTTTAATTAAAGAATTTGAATGGCAGAAGATAAACGCCTAG<br><br>>Translation of ORF number 1 in reading frame 1 on the direct strand.<br><br>EEDHSVTERFGKSPDLRQCGQSLIKEFEWQKINA*	Ter after 35 AA<br><br>p.Asp142GluTer35
**c.423+294G>T**<br><br>**Transcript 45b**	r.423_424ins[423+161_423+288]	Exon 4 end: CCC (Pro 141)<br><br>GAGGAGGACCACAGTGTCACTGAGAGATTAGGAAAATCTCCAGATCTGAGACAGTGTGGCCAGAGTTTAATTAAAGAATTTGAAT	>ORF number 1 in reading frame 1 on the direct strand extends from base 1 to base 105.<br>GAGGAGGACCACAGTGTCACTGAGAGATTAGGAAA	Ter after 35 AA<br><br>p.Asp142GluTer35

		GGCAGAAGATAAACGCCTAGAAAATCCTCCAGCAGCTCATTTG<br><br>Exon 5 beginning: GAT (Asp 142)	ATCTCCAGATCTGAGACAGTGTGGC<br>CAGAGTTTAATTAAAGAATTTGAATGGCAGAAGATAAACGCCTAG<br><br>>Translation of ORF number 1 in reading frame 1 on the direct strand.<br><br>EEDHSVTERLGKSPDLRQCGQSLIKEFEWQKINA*	
**c.423+296T>A**<br><br>**Transcript 46b**	r.423_424ins[423+161_423+293]	Exon 4 end: CCC (Pro 141)<br><br>GAGGAGGACCACAGTGTCACTGAGAGATTAGGAAAATCTCCAGATCTGAGACAGTGTGGCCAGAGTTTAATTAAAGAATTTGAATGGCAGAAGATAAACGCCTAGAAAATCCTCCAGCAGCTCATTTGGTGAG<br><br>Exon 5 beginning: GAT (Asp 142)	>ORF number 1 in reading frame 1 on the direct strand extends from base 1 to base 105.<br>GAGGAGGACCACAGTGTCACTGAGAGATTAGGAAAATCTCCAGATCTGAGACAGTGTGGC<br>CAGAGTTTAATTAAAGAATTTGAATGGCAGAAGATAAACGCCTAG<br><br>>Translation of ORF number 1 in reading frame 1 on the direct strand.<br><br>EEDHSVTERLGKSPDLRQCGQSLIKEFEWQKINA*	Ter after 35 AA<br><br>p.Asp142GluTer35
**c.200+5G>C**<br><br>**Transcript 8g**	r.201_286delins200+1_200+236	Exon 2 end: AA (Lys 67)<br><br>GTGAGTATCTCTGTGAAAGCCAGCTATTGAAGGAGAGTTCTTGATTTGATTTAGGGACATGCTTTTCACATCCTTGGAAGGCTTAAAAATCTGAGATCATCCGATGGCTCTGTTTTTAAAACAAGAATGAGCTTGGGCCGGGCACGGTGGCTCACGCCTATAATCCCAGCACTTTGGGAGGCTCAGGCGGGCAGATCATCTGAGGTTGGGAGTTCAAGACCAATGTGGCCAACATG<br><br>Exon 4 beginning: G (Next base due to Exon 3 skipping)<br><br>Exon 3 beginning: A TGT (Cys 68)	>ORF number 1 in reading frame 1 on the direct strand extends from base 1 to base 6.<br>AAGTGA<br><br>>Translation of ORF number 1 in reading frame 1 on the direct strand.<br>K*	p.Cys68Ter
**c.200+5G>C**<br><br>**Transcript 8c**	r.200_201ins[200+1_200+30; 201-2_201-1] -> 200+5 G>C included?	Exon 2 end: AA (Lys 67)<br><br>GTGACTATCTCTGTGAAAGCCAGCTATTGA<br><br>Exon 3 beginning: A TGT (Cys 68)	>ORF number 1 in reading frame 1 on the direct strand extends from base 1 to base 6.<br>AAGTGA<br><br>>Translation of ORF number 1 in reading frame 1 on the direct strand.<br><br>K*	p.Cys68Ter
**c.200+1G>A**<br><br>**Transcript 2d**	r.200_201ins[a; 200+2_200+30; 201-2_201-1]	Exon 2 end: AA (Lys 67)<br><br>ATGACTATCTCTGTGAAAGCCAGCTATTGA<br><br>Exon 3 beginning: A TGT (Cys 68)	>ORF number 1 in reading frame 1 on the direct strand extends from base 1 to base 6.<br>AAATGA<br><br>>Translation of ORF number 1 in reading frame 1 on the direct strand.<br><br>K*	p.Cys68Ter
**c.287-1G>T (Transcript 26e)**	r.201_423del	Exon 2 end: AA (Lys 67)<br><br>Exon 3 beginning: A TGT (Cys 68)<br><br>Exon 5 beginning: GAT (Asp 142)	>ORF number 1 in reading frame 1 on the direct strand extends from base 1 to base 9.<br><br>AAGATTTGA	p.Cys68IleTer2

**c.287-1G>C (Transcript 58b)**<br><br>**c.287-2A>T (Transcript 3b)**<br><br>**c.287-3C>G (Transcript 25c)**<br><br>**c.287-5T>G (Transcript 24c)**		Exon 5 sequence:<br><br>(GAT)TTGAGCAACTTCTATGCACAGTACAAATCCATTGAGCCTTATTTGAAGAAGAAGGA	>Translation of ORF number 1 in reading frame 1 on the direct strand.<br>KI*	
**c.200+5G>C (Transcript 8f)**	r.73_286del	Exon 1 end: CAG (Gln 24)<br><br>Exon 2 beginning: GCC (Ala 25) S26 R27<br><br>Exon 4 beginning: GC<br><br>Exon 4 sequence:<br>(GC)ATCTGTGGCTCTTGTGCAATGAACATCAATGGAGGCAACACTCTAGCTTGCACCCGAAGGATTGACACCAACCTCAATAAGGTCTCAAAAATCTACCCTCTTCCACACATGTATGTGATAAAGGATCTTGTTCCC	>ORF number 1 in reading frame 1 on the direct strand extends from base 1 to base 24.<br>GCATCTGTGGCTCTTGTGCAATGA<br><br>>Translation of ORF number 1 in reading frame 1 on the direct strand.<br><br>ASVALVQ*	p.Arg27ValTer6

Supplementary References

1. Abou Tayoun, A. N. *et al.* Recommendations for interpreting the loss of function PVS1 ACMG/AMP variant criterion. *Hum. Mutat.* **39**, 1517–1524 (2018).
2. Parsons, M. T. *et al.* Evidence-based recommendations for gene-specific ACMG/AMP variant classification from the ClinGen ENIGMA BRCA1 and BRCA2 Variant Curation Expert Panel. *Am. J. Hum. Genet.* **111**, 2044–2058 (2024).
